# Reconstructing spruce budworm outbreak severity: a comparison of paleoecological and tree-ring signals

**DOI:** 10.1371/journal.pone.0329406

**Published:** 2025-08-12

**Authors:** Marc-Antoine Leclerc, Martin Simard, Hubert Morin

**Affiliations:** 1 Department of Fundamental Sciences, Université du Québec à Chicoutimi, Chicoutimi, Québec, Canada; 2 Department of Geography, Université Laval, Québec City, Québec, Canada; Technical University in Zvolen, SLOVAKIA

## Abstract

Applying a centennial or millennial perspective to disturbance regimes permits an understanding of how these events have varied in the past in relation to climate change. Correctly interpreting this variability is crucial when preparing sustainable forest management practices for future warming. The eastern spruce budworm (Lepidoptera) is the most important biotic disturbance in the eastern Canadian boreal forest. Adult moths are covered by chitinous scales, and lepidopteran scale records in lake sediments have been analyzed to reconstruct Holocene spruce budworm populations. However, the magnitude of these scale accumulations has yet to be calibrated using an independent proxy. Here, we determine whether the impacts of spruce budworm defoliation are recorded by both sedimentary lepidopteran scale accumulations and tree-ring widths. Agreement between proxies was found at five of nine sites and strongest between the proportion of affected trees and scale accumulations while agreement in signal synchronicity was found at six of nine sites and strongest when comparing scale accumulations to a growth suppression index. A species-based composite chronology relying on white spruce produced the clearest outbreak record for both proxy records. Peak scale accumulations correlated well with smaller tree-ring widths, demonstrating that larger scale accumulations correspond to more severe defoliation events. Therefore, lepidopteran scales provide reliable records of spruce budworm abundance serving as a proxy record ameliorating our understanding of how budworm impacts have fluctuated at centennial and millennial time scales in the context of past climate change.

## Introduction

Climate change is expected to favour more frequent and severe disturbance events [1,2]. In the boreal biome, potentially affected disturbance regimes include insect outbreaks, as insect survival, growth, and development are influenced directly or indirectly by a changing climate. The projected climate-related consequences include the direct effects of temperature change [3–5], modified insect and host-tree distributions [6,7], feeding on novel host-tree species [8–10], and an altered insect-host tree phenology, possibly rendering less vulnerable hosts more vulnerable [11–13]. Indirect climate effects, via drought and variable precipitation regimes, could result in greater host-tree mortality in conjunction with insect outbreaks [14–18], or a loss in outbreak regularity [19].

In the eastern Canadian boreal forest, the spruce budworm [*Choristoneura fumiferana* Clemens], a native lepidopteran defoliator experiencing episodic outbreaks, is the main biotic disturbance [20,21]. The spruce budworm completes its life cycle in a single year beginning as an egg, molting through six larval stages, forming a pupa, and emerging as an adult moth that can then reproduce and disperse [21–24]. Defoliation by larvae occurs preferentially on the current year needles of mature balsam fir [*Abies balsamea* (L.) Mill.], with older needles targeted at high populations densities [21,25,26]. Meanwhile, white [*Picea glauca* (Moench) Voss] and black [*Picea mariana* (Mill.) Britton, Sterns & Poggennburg] spruce serve as alternate budworm hosts [27]. Defoliation by the larvae often results in growth reductions [28–31], and severe defoliation will result in tree mortality, particularly in the preferred host [32,33] as opposed to black spruce which has historically experienced lower levels of defoliation [34] but this may no longer be the case given climate change [5,11–13]. Twentieth-century outbreaks of this insect in eastern Canada have occurred roughly every 30–40 years [31,35–37] varying in duration [38] and severity [32,39–41] producing important consequences for timber supply, and carbon cycling [42].

Spruce budworm outbreaks over the last 300–400 years have been reconstructed using dendrochronology with the detection and severity of such events identified by narrow tree-ring widths [29–31,35,36,43–45]. Narrower ring widths reflect a lagged signal [46–48] of inhibited growth due to the reduction in a tree’s photosynthetic capacity [46,49], acting as a proxy of abundant spruce budworm and its feeding on needles, i.e., defoliation, once the background climate signal is removed [50]. However, the accuracy of dendrochronological records of outbreaks can depend on the tree species cored [29,30,51–53], surviving individuals of past disturbance events and their ability to record outbreaks [54] i.e., survivor bias, and is limited by a tree’s lifespan [55] resulting in a fading record by biasing reconstructions to more recent events [53,56]. As such, dendrochronological reconstructions of spruce budworm outbreaks at millennial or multi-millennial scales are limited [57].

Therefore, the use of other proxy records is required to extend spruce budworm outbreak reconstructions over thousands of years. Previous paleoecological approaches to identify spruce budworm outbreaks have included sediment records of larval head capsules, and their feces but these proxies exhibited low abundance and degradation issues, respectively [58,59]. More recently, chitinous lepidopteran scales, which cover the myriads of adult moths present during an outbreak, have proven to be an excellent proxy of spruce budworm populations [60,61]. The abundance of scales in the sedimentary record—thousands cover a single adult budworm moth—and their long-term preservation in the lake sediment record related to their chitinous composition [62–64] improve on the former insect outbreak proxies. Moreover, the abundance of scales deposited into small lakes mirrors the local insect biomass, and lepidopteran scales are incorporated into the lacustrine sediment within the year [65]. Finally, there is a high agreement between the photographic aerial survey record (1967-present) and the abundance peaks in the lepidopteran scale sedimentary record [66].

Despite the potential of scales to track long-term spruce budworm abundance, further calibration remains. Here, we compare local dendrochronological and corresponding lepidopteran scale sedimentary records from a series of lakes. We aim to establish whether both proxy records record similar impacts of spruce budworm defoliation and, if so, assess signal synchronicity. We posit two hypotheses: 1) larger accumulations of lepidopteran scales in the sedimentary records should match tree-rings records showing growth suppression/greater percentage of affected trees because greater scale accumulations in the sediment record should track larger adult moth populations around the lake and more severe defoliation by larvae; and 2) the signals from the sedimentary and tree-ring records should be relatively synchronous because lepidopteran scale incorporation into the sediment is relatively quick [65], and the effects of defoliation as recorded by tree-ring widths become evident two to four years following a defoliation event [47,48].

## Methods

### Ethics statement

This study was conducted in Québec’s unprotected public lands and permission was obtained from Québec’s Ministry of Forest, Fauna and Parks, the governing body managing these lands, and field work did not require a permit. No protected or endangered species were used or sampled in the course of this research.

### Lepidopteran scales]

#### Field sampling, subsample preparation, and identification.

The same sites, surface sediment samples, age-depth models, and methods described in Leclerc et al. [66] were used to obtain sedimentary lepidopteran scale records of spruce budworm outbreaks. Briefly, a gravity corer [67,68] sampled the water-sediment interface of nine lakes located at sites that have recently sustained variable levels of defoliation [66] (Table 1). Sediment cores were sampled at a 1 cm resolution and scale recovery and analysis followed the protocol outlined in Leclerc et al. [66]. Finally, the age-depth models using ^210^Pb activity derived from the constant rate of supply model [69,70] were the same as those published in Leclerc et al. [66].

**Table 1:** Characteristics of the sampled lakes and of their surrounding forest.

			Forest composition (%)^1^				Forest disturbance history^2^		
Lake (North to South)	Latitude Longitude	Lake area (ha)	Spruce *spp.*	Balsam fir	Jack pine	Decid.	Fire	Forest harvest	Defoliation
5	49.6070 −71.9980	3.91	17	51	2	12	1890	1996-1997, 2018	S
8	49.4454 −71.3322	5.37	3	39	–	45	–	< 1970, 1990	L
2	49.3648 −72.0296	4.11	34	2	3	18	–	< 1970, 1991, 1993, 2010	S
1	49.3200 −72.2860	1.20	58	–	9	–	1890, 1940	1995, 2016	S
3	49,0120 −71.6460	2.62	15	21	–	24	–	< 1970, 1982, 1993, 1997, 2014, 2017	M
4	48.8718 −71.5664	0.94	39	–	20	2	1951, 1985	< 1970	L
Bois Joli	48.2451 −71.2419	0.90	–	11	–	69	1900	< 1970, 1986, 1988, 2013	M
Buire	48.1654 −70.5708	1.30	18	11	–	60	–	< 1970	M
Bélanger	47.4765 −75.1775	2.79	10	13	–	53	–	< 1970	–

All sediment cores were collected in 2019 except for Lakes 4 (2018), Buire (2018), and Bélanger (2017).

^1^ Source: Fourth decadal forest inventory, Québec Ministry of Forests, Fauna, and Parks [71]. Because these maps are based on photo-interpretation, spruce species (white spruce [*Picea glauca*] and black spruce [*Picea mariana*]) are not distinguished. Decid.: deciduous species (mainly trembling aspen [*Populus tremuloides*] and white birch [*Betula papyrifera*]).

^2^ Source: Fire and forest harvest history obtained from the Fourth decadal forest inventory [71]. Spruce budworm defoliation history obtained from aerial surveys of the Québec Ministry of Forests, Fauna, and Parks [72]. Defoliation severity during the year of sampling is L: light, M: moderate, S: severe, and -: no defoliation detected.

### Tree-ring record for the 1900–2019 period

#### Field sampling, preparation, and identification.

We produced local chronologies at each site by coring multiple trees (20–32 of the largest and oldest individuals) within a 200 m radius around each lake (from herein ‘site’ will refer to the sediment collected from each lake and the respective cored trees) but up to an 800 m radius to sample enough individuals. We extracted one to two cores from each tree at a height of approximately ≤30 cm above the ground surface, or in certain cases ≤70 cm above the ground surface depending on the presence of rot in the stem. Cores were prepared and mounted following standard dendrochronological procedures [73]. Cores were scanned, and ring widths were measured using WinDendro software [74,75]. Samples were visually cross-dated using PAST5 software [76] guided by the Pearson correlation coefficient, threshold values of 60%–70% for the ‘Gleichlauf test’, and ≥4 for the Baillie/Pilcher and Hollstein t-tests to ensure correct dating [77]. We subsequently verified cross-dated sample placement via COFECHA v.6.06 [78] to confirm visual cross-dating. We then used chronology stripping [79] and the expressed population signal (EPS) [80,81] to select individual trees that would make up the most representative chronology for the 1900–2019 period for each site in the *dplR* package [82]. These selected individual trees were detrended and standardized with a 60-year cubic spline in *dplR* [82] to remove any low frequency signals [36,83–86], and highlight higher frequency changes that would result from spruce budworm defoliation to obtain ring-width indices, and finally produce the most representative site chronologies. The reliability of these chronologies over the study period (1900–2019) was assessed using Subsample Signal Strength with a threshold value of 0.85 [80]. These and all subsequent analyses were performed in the R statistical environment (v.4.0.5) [87].

Defoliation events described by the mean growth suppression index (GSI), and proportion of defoliated trees (‘percent affected’) for each site were obtained using the R package *dfoliatR* [88] using the methods and criteria developed by Swetnam et al. [50]. Defoliation events were first identified using the *defoliate_trees* function with default parameters [50,88] but adjusting the *bridging events* and *series-end events* to capture the current outbreak and avoid detecting multiple defoliation events that were likely part of a single event [88–90]. We calculated each site’s mean GSI and percent affected at an annual resolution with the *outbreak* function using default settings [88–90]. The tree-level GSI is usually calculated as [50,88,89]:


$${GSI}_{i}=H_{i}-({NH}_{i}-{NH}_{m})\frac{\sigma_{H}}{\sigma_{NH}}$$ (1)

where GSI_i_ is the growth suppression index in year *i*, H and NH are the standardized host-tree and non-host ring-width series, respectively, NH_m_ is the mean non-host series’ ring-width, and $\frac{\sigma_{H}}{\sigma_{NH}}$ is the ratio between the standard deviation of the standardized host and non-host series [50,88]. In this study, lack of local non-host individuals precluded the creation of a non-host series and use of a non-host correction such that the GSI_i_ simply became the standardized host-tree ring-width series:


$${GSI}_{i}=H_{i}$$ (2)

In lieu of a non-host correction, the Standardized Precipitation Evapotranspiration Index (SPEI) [91,92] from 1901–2011 [93] was used to confirm that growth reductions recorded by the tree-rings were in fact due to defoliation by the spruce budworm (S1 Appendix).

### Correlation and synchronicity between scale and tree-ring records

The correlation and synchronicity between lepidopteran scale and tree-ring records (mean GSI and percent affected) were assessed using wavelet analysis. First, lepidopteran scale accumulations over the entire sediment chronology for each site were interpolated to an annual resolution by determining the relative proportion that the sediment subsample(s) contributed to each respective year and then weighing the subsample(s) based on these respective proportions to obtain an accumulation for each year [94] using the *pretreatment* function from the *paleofire* R package [95] to retain the variability in these accumulations. The interpolation permitted directly comparing scale accumulation rate, mean GSI, and percent affected. Further, interpolation to a constant time step attempted to reduce potential effects of sediment compaction that could have occurred in the scale records. The signal of each variable for each site was modelled using generalized additive models (GAMs) with a penalized cubic regression spline in the *mgcv* package [96]. Model fit was evaluated with the *gam_check* function by assessing the distribution of the residuals, normality, and the relationship between the measured and predicted variables [96].

Lake 8 required removing and imputing a point (CE 1952) in the mean GSI data set to better model the mean GSI signal and ensure the constant time step required for wavelet analysis. The comparison between the original and altered data set is presented in S2 Appendix.

The correlation and synchronicity between the lepidopteran scale and mean GSI signals and between the scale and the percent affected signals were assessed using wavelets with the *biwavelet* R package [97] over the time period (1900–2019) common to both records as the trees cored were not old enough to go further back in time. The use of wavelets is particularly powerful for analyzing and comparing non-stationary signals through time [98–102]. We applied a continuous Morlet wavelet transform, using default settings [97], to obtain the temporal trends for each variable because this particular continuous wavelet transform is robust to noise, can extract phase interaction information between a pair of time series [99,100,103], offers a relatively good balance between time and frequency [99], and has a high frequency resolution [100] allowing for precise and accurate identification of spruce budworm impact signals. For each site, we tested the strength of each variable’s signal (significance in the power spectrum) against a background signal generated by an autoregressive process with a lag of 1, using a $\chi^{2}$ test and an α value of 0.05 [97,98].

Subsequently, we assessed any correlations between the scale and tree-ring records in the *biwavelet* R package via cross-wavelet analysis and wavelet coherence running 1000 Monte Carlo randomizations for the latter [97]. Cross-wavelet analysis reveals points in time and return intervals (i.e., periodicities) where strong signals between the variables overlap, and which signal leads or lags [99,104]. In the time–frequency dimension, wavelet coherence identifies correlations between a pair of measured series as a function of frequency, with the correlation ideally displaying a consistent relationship between signals, i.e., phased-locked behaviour [98–100,105]. The significance for both wavelet coherence and cross-wavelet analysis was tested against a background signal generated by an autoregressive process with a lag of 1, using a $\chi^{2}$ test, and an α value of 0.05.

Finally, we determined the nature of the interaction between the two proxies by determining the phasing of the signal pairs, that is, which signal was leading or lagging relative to the other [99,100]. Phasing was key in testing the posited hypotheses by quantifying the agreement/disagreement between the proxy record signals (see below) ultimately determining whether lepidopteran scales were a reliable recorder of spruce budworm outbreaks. Phasing was represented graphically by arrows overlaid on wavelet power spectra with the arrowhead direction indicating the nature of the interaction. Arrows pointing to the right indicate that the two signals are in-phase when the peaks and troughs of both signals are synchronous. An arrow pointing to the left indicates an anti-phase relationship when the peak of a signal lines up with the trough of the other signal [99]. An upward arrow indicates that the scale record lags the dendrochronological record by $\frac{\pi }{2}$ [99] i.e., a peak/trough in lepidopteran scales occurs slightly *after* a peak/trough in either the mean GSI or percent affected. A downward arrow indicates that the scale record leads the tree-ring record by $\frac{\pi }{2}$, i.e., a peak/trough in lepidopteran scales occurs slightly *before* a peak/trough in either the mean GSI or percent affected [99]. We calculated the circular mean and standard deviation using the *circular* R package [106] for the entire zone of significant high overlapping power and in zones of high correlation (R^2^ ≥ 0.80) over the entire study period (1900–2019) and for each known outbreak period (1912–1929, O3; 1946–1959, O2; and 1975–1992, O1) [35,36,43] for each site.

Using the circular mean values and standard deviation, we described the signals as being in agreement (lepidopteran scale and mean GSI signals showing an anti-phase behaviour) or disagreement (in-phase behaviour represents disagreement). We expect large spruce budworm impacts, related to large adult moth populations (abundant scale accumulations), will be reflected by a lower mean GSI (narrower tree-ring widths) because of greater larval defoliation. Conversely, lepidopteran scale and percent affected signals agree when these records exhibit in-phase behaviour and disagree when exhibiting anti-phase behaviour.

### Composite chronologies

We created composite lepidopteran scale and tree-ring chronologies to determine whether a clearer signal could be obtained by combining sites and basing these composite records on tree species (three white spruce and six black spruce sites). We combined standardized individual tree-level ring widths from the three white spruce sites to obtain the white spruce composite. In contrast, the black spruce composite was built using the standardized individual tree-ring widths from the six black spruce sites. The respective ring-width and percent affected composite records were developed using the *dplR* [82], and *dfoliatR* [88] packages respectively. We combined the annually interpolated lepidopteran scale accumulations, obtained via the *pretreatment* function in the R *paleofire* package [95], from the respective white and black spruce sites to obtain a composite mean lepidopteran scale accumulation for each tree taxa.

## Results

### Age-depths models, and lepidopteran scale and tree-ring proxy records

The age-depth models derived from ^210^Pb measurements used to determine lepidopteran scale accumulations displayed similar shapes and low variability in date estimates over the study period (1900–2019; supplementary material in [66]). Lepidopteran scale accumulations, ring-width indices, and percent trees affected varied from site to site (S3 Appendix).

### Wavelet analyses

**Fig 1:**
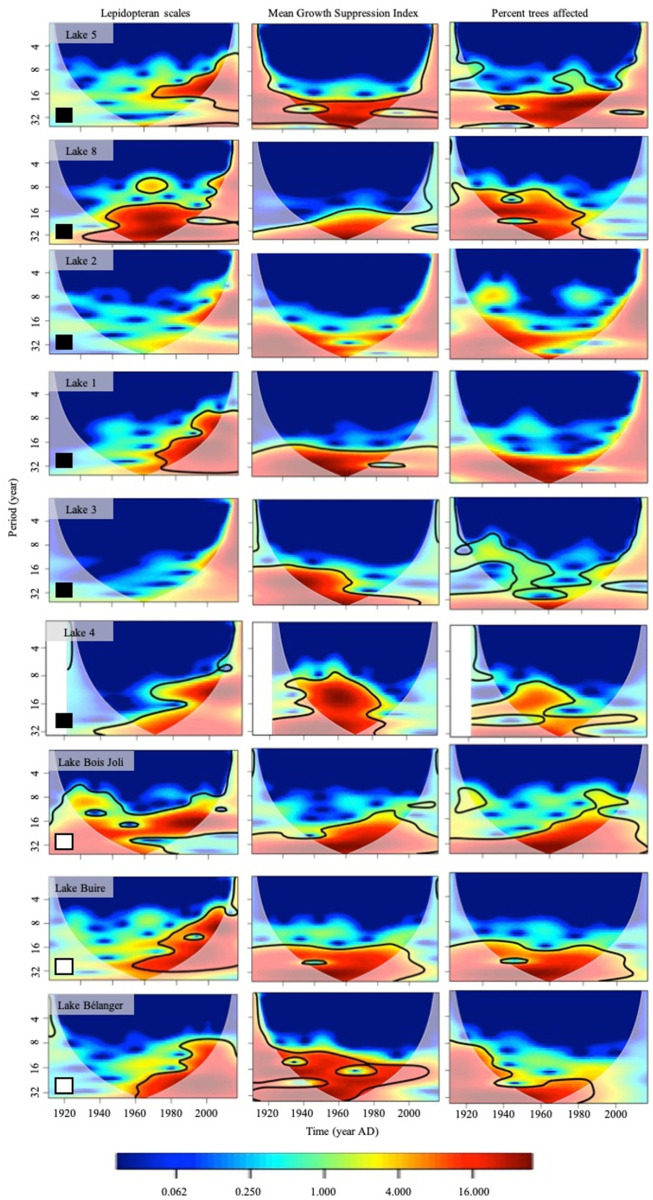
The power spectra of the paleo and tree-ring variables from 1900 to 2019. Warmer colours (red, orange, yellow) indicate high power relative to the background signal generated by an autoregressive process with a lag of 1, whereas cooler colours represent weaker power [98]. Statistically significant zones of power were determined using a $\chi^{2}$ test (p < 0.05) and are delineated by a thick black line [98]. The light grey shading delineates the recommended zone of interpretation, i.e., the cone of influence [100]. Squares in the left-hand corners of the spectra indicate the tree species cored; white, white spruce; black, black spruce.

The individual continuous Morlet wavelet transforms revealed strong significant return intervals (periodicities) in the scale and tree-ring records (Fig 1). Areas of significant high power in lepidopteran scale abundance had a periodicity of 8–32 years in seven out of nine sites from 1950–2019 (Fig 1). Areas of significant high power in the mean GSI, and percent affected also had a similar periodicity (8–32 years) for most of the study period (1900–2019) in seven of the nine sites (Fig 1). We observed areas of high power for mean GSI over the entire study period at all sites except Site 2 and high power for percent affected over the entire study period at all sites except Sites 1, and 2 (Fig 1).

**Fig 2:**
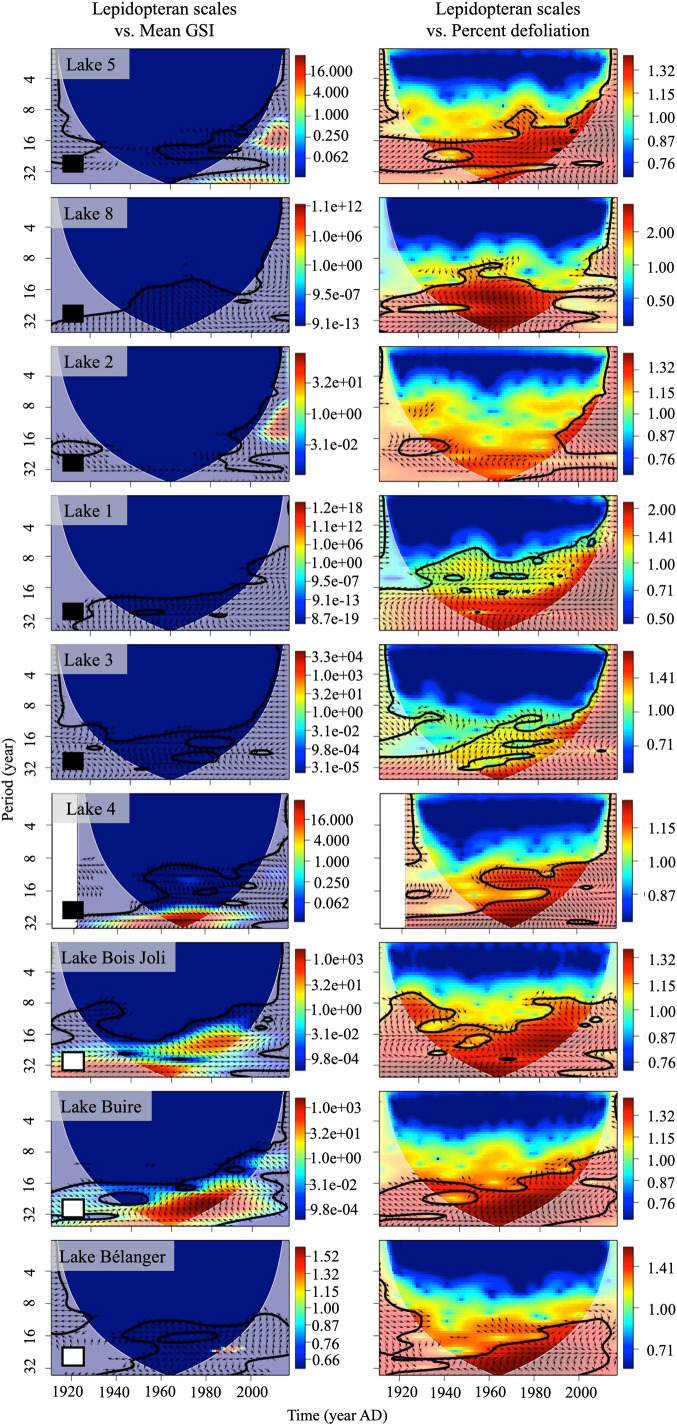
The power spectra resulting from the cross-wavelet analysis applied to the paleo and dendrochronological records. Warmer colours (red, orange, yellow) indicate high common power relative to a background signal generated by an autoregressive process with a lag of 1, whereas cooler colours represent weaker power [98]. Statistically significant zones of common power were determined using a $\chi^{2}$ test (p < 0.05) and are delineated by a thick black line [98]. The arrows in the areas of statistical significance help specify the type of association. Arrows pointing left indicate that the signals are anti-phase, where a peak of one signal lines up with a trough of the other signal. Arrows pointing right suggest that signals are in-phase, where peaks and troughs of both signals line up. Downward arrows indicate that the scale record leads the tree-ring record by $\frac{\pi }{2}$, whereas upward arrows indicate the opposite. The light grey shading delineates the recommended zone of interpretation, i.e., the cone of influence [100]. Squares in the left-hand corners of the spectra indicate the tree species cored; white, white spruce; black, black spruce.

The cross-wavelet analysis between both proxies revealed a stronger signal between lepidopteran scales and the percent trees affected than between lepidopteran scales and the mean GSI at an 8-to-32-year periodicity over the entire study period for all sites (Fig 2; Table 2). Agreement at five of nine sites was observed in both lepidopteran scale-mean GSI, and lepidopteran scales-percent affected comparisons. Further consistent agreement between records was observed at Sites 2, 3, 4, and Bélanger for both comparisons (Fig 2; Table 2), noting however, that phases were variable within the zones of significant overlapping power, and also among sites.

**Table 2:** Characteristics of tree-ring chronologies and wavelet analysis results comparing spruce budworm outbreak proxies.

			Wavelet analyses															
			Cross-wavelet								Wavelet coherence							
	Tree-ring chronologies		Scales vs. GSI				Scales vs. Pct Aff				Scales vs. GSI				Scales vs. Pct Aff			
Lake (North to South)	Species (*N* trees)^1^	EPS^2^	All	O3	O2	O1	All	O3	O2	O1	All	O3	O2	O1	All	O3	O2	O1
5	BSp (23)	0.931 (1900-2019)	A	A	D	D	D	A	D	D	A	A	A	A	A	D	A	A
8	BSp (17)	0.750 (1969-2019)	D	A	D	D	A	A	A	A	D	D	D	D	D	D	D	D
2	BSp (27)	0.909 (1931-2019)	A	D	–	D	A	D	–	A	A	A	A	A	A	A	A	A
1	BSp (24)	0.874 (1915-2019)	D	D	D	D	D	A	D	D	A	A	A	A	A	A	A	A
3	BSp (27)	0.895 (1942-2019)	A	D	A	A	A	A	A	A	A	A	A	A	A	A	A	A
4	BSp (31)	0.986 (1960-2019)	A	–	A	A	A	–	A	A	D	–	–	D	D	–	A	D
Bois Joli	WSp (27)	0.885 (1912-2018)	D	D	D	D	D	D	D	D	D	D	D	D	D	D	N/A	N/A
Buire	WSp (25)	0.861 (1900-2018)	D	D	A	D	D	D	A	D	A	D	A	A	D	D	D	D
Bélanger	WSp (26)	0.963 (1942-2019)	A	A	D	D	A	A	D	A	A	A	A	A	A	A	D	A

The agreement (A) or disagreement (D) between paleoecological (Lepidopteran scales) and tree-ring (Growth Suppression Index [GSI] or Percent trees affected [Pct aff]) records was assessed for the last three spruce budworm outbreaks (O1: 1975–1992, O2: 1946–1959, O3: 1912–1929) and for the whole period (All: 1900–2019) common to both records. Agreement could not be determined for some outbreaks (indicated by “-”) because of lack of data (Lake 4, O3) or when relationships were not significant.

^1^ BSp: black spruce, WSp: white spruce. *N trees*: number of trees included in the chronology

^2^ Expressed Population Signal of the most representative chronology for each site over the entire study period (1900–2019) obtained via chronology stripping [79]. Brackets indicate the periods where Subsample Signal Strength was > 0.85.

**Fig 3:**
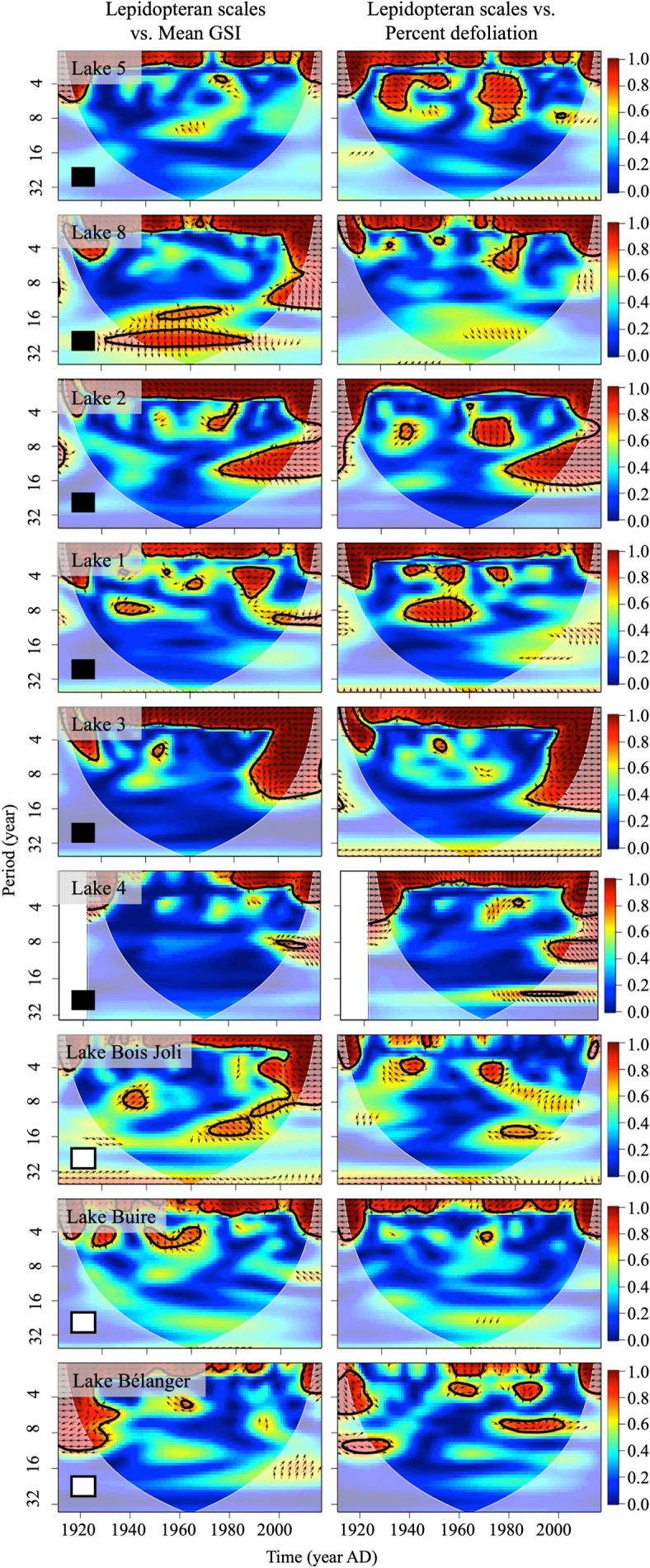
The power spectra of the wavelet coherence analysis applied to the paleo and dendrochronological records. Comparison between the lepidopteran scale and mean GSI time series and the lepidopteran scale and percent affected time series. Warmer colours (red, orange, yellow) indicate high correlation relative to a background signal generated by an autoregressive process of a lag of 1, whereas cooler colours represent weaker correlation [98]. Statistically significant zones were determined using a $\chi^{2}$ test (p < 0.05) and are delineated by a thick black line [98]. The arrows in the areas of statistical significance help specify the type of association. Arrows pointing left indicate that the signals are anti-phase, where a peak of one signal lines up with a trough of the other signal. Arrows pointing right suggest signals are in-phase, where peaks and troughs of both signals line up. Downward arrows indicate that the scale record leads the tree-ring record by $\frac{\pi }{2}$, whereas upward arrows suggest the opposite. The light grey shading delineates the recommended zone of interpretation, i.e., the cone of influence [100]. Squares in the left-hand corners of the spectra indicate the tree species cored; white, white spruce; black, black spruce.

We observed a highly significant wavelet coherence between the scale and tree-ring records at a one-to-four-year periodicity over the study period (Fig 3). Agreement between records was observed at six, and five of nine sites for scale-mean GSI, and scale-percent trees affected comparisons in the zones of high correlation (R^2 ^≥ 0.80) respectively (Fig 3; Table 2). Further, consistent agreement was observed at Sites 1, 2, 3, 5, and Bélanger (Table 2) suggesting that greater scale accumulations coincided with narrower ring widths or a greater proportion of affected trees. Significant correlations observed at higher periodicities were noteworthy at Sites 2, 3, and 8 (Fig 3).

Finally, agreement between both proxies at the individual outbreak level varied between sites and depended on the applied analysis (Table 2). We observed a more consistent outcome for the wavelet coherence analysis, although this consistency was site dependent (Table 2). For example, we found consistent agreement and disagreement in wavelet coherence between both proxies for all outbreaks at three sites and one site respectively. Agreement for cross-wavelet analysis at the outbreak level was more variable, and only Lake Bois Joli displayed a constant disagreement between both proxies for all outbreaks (Table 2).

### Composite chronologies

**Fig 4:**
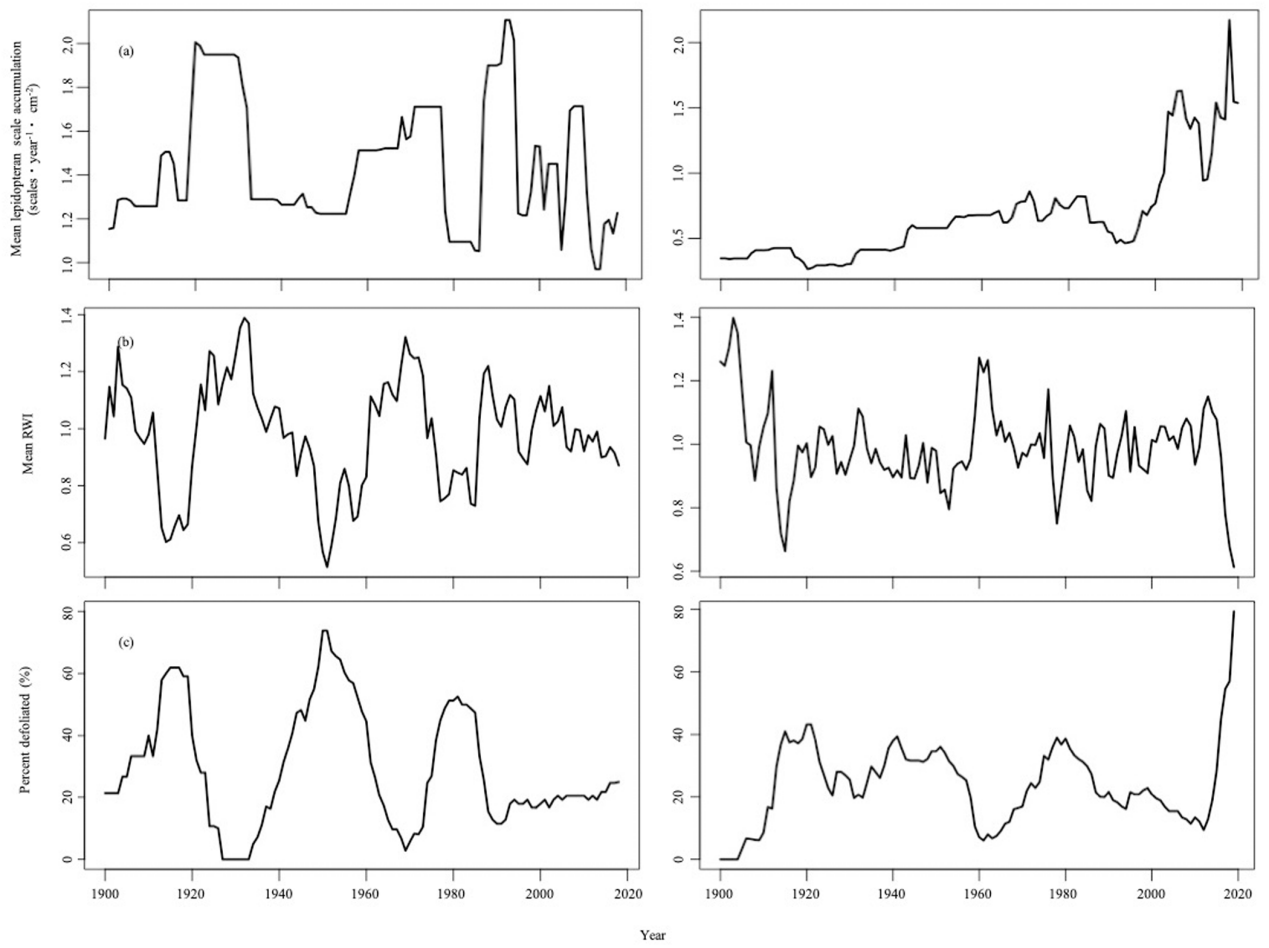
The white and black spruce composite chronologies of the scale and tree-ring records. The white (left column) and black (right column) spruce records comprise (A) the mean lepidopteran scale accumulation, (B) the mean ring-width index (RWI), and (C) the proportion of affected trees (percent affected). The white spruce composite incorporates data from three sites. The black spruce composite incorporates data from six sites.

By creating species-specific composite chronologies, we obtained a stronger, and clearer signal of spruce budworm impact for both proxies in the white spruce composite relative to the black spruce composite. The white spruce composite of lepidopteran scale accumulation revealed three relatively distinct peak scale accumulations corresponding to the three 20^th^ century outbreaks (Fig 4). The ring-width index revealed three clear areas of growth suppression with three corresponding peaks in the proportion of affected trees. We did observe, however, a consistent 10-year lag between the scale and tree-ring records. We only observed a single large peak scale accumulation in the black spruce composite from ~2000 AD (Fig 4). Similarly, the dendrochronological black spruce composite revealed a major growth suppression and an increased proportion of affected trees from ~2000 AD to 2019 (Fig 4). The variability in the black spruce composite proxy records was such that the other outbreak signals were difficult to identify (Fig 4) suggesting more variable levels of defoliation and/or a more variable ability to record defoliation by this species.

## Discussion

### Proxy record strengths, and limitations

Both the scale and tree-ring records record the impact of spruce budworm defoliation, and the signals are relatively synchronous, validating the use of lepidopteran scales for reconstructing centennial to multi-millennial spruce budworm population fluctuations. Lepidopteran scales are assumed to be a direct measure of the size of the adult moth spruce budworm population as their count in the sediment represents the changing insect biomass at a site over time (a stand-level phenomenon), with a greater count at a particular depth reflecting a larger budworm population. This assumption is based on prior knowledge of the insect’s biology, including life cycle and development, feeding behaviour and impacts [21,23,107–109], and the epidemiology of past outbreaks [31,36,44,54,110]. In addition to representing changes in adult spruce budworm populations, lepidopteran scale accumulations are correlated with severe annual defoliation [65], indicating that greater accumulations may reflect more severe events. However, despite a relatively quick incorporation into the sediment record [63,65], the lepidopteran scale signal is affected by the rate of sediment deposition and mixing both highly variable among lakes, and even within a given lake [111]. There is also error associated with the dating of the sediment record [112] producing artificial lags or asynchrony between the sediment record and the tree-ring record.

The tree-ring record, from which the mean GSI and percent affected variables are derived, is an indirect indicator of the spruce budworm presence. Specifically, mean GSI requires the growth records of several individual trees to obtain a mean stand-level growth pattern. An individual tree’s reaction, i.e., the production of narrower growth rings, to a defoliation event is nonetheless unique in that multiple contributing factors, such as site conditions [113], species composition [39,40,114,115], genetics [116,117], age [33,118], and tree height [119,120] can influence the extent, and speed that a tree exhibits growth suppression [41,46,121]. The reaction and response time can thus introduce variation in the mean stand growth to obscure the spruce budworm signal.

The percent affected metric, also based on tree ring widths, reflects the proportion of trees affected by the spruce budworm. More specifically, it is binary; depending on the applied defoliation threshold, a tree is counted as having been defoliated or not [88]. Contrary to the mean GSI, which quantifies the change in the mean stand-level growth over time, percent affected is a coarser measure of the spruce budworm’s impact on the stand because it testifies to changes in the proportion of trees considered as defoliated over time [88]. However, both are based on the survivors of past outbreaks and other disturbances. Therefore, these survivors of past defoliation events may not necessarily reflect the true severity of the event [53,54,83]. Moreover, harvesting and wildfires may remove trees that likely recorded a spruce budworm signal. Those remaining trees tend to be younger and typically less affected by defoliation [32,33,118]. Finally, the tree species may influence the recorded spruce budworm signal. Defoliation on white spruce is reliably recorded [5,29,30,51], whereas the defoliation signal in black spruce may not be as clear [28,30] despite their use in reconstructing outbreaks [48,52]. Therefore, it is important to note that although the tree-ring record is more temporally precise than the paleo proxy, recorded annual changes in growth depend on survivors, and incorporates large amounts of variability such that annual changes in local defoliator populations cannot be directly inferred from this record [122].

### Proxy record signal comparison, and factors affecting proxy signals

Both the scale and tree-ring records highlighted strong significant return intervals (periodicities) in spruce budworm populations. The strong return interval signal identified by both proxies typically ranged from 16 to 32 years over the study period likely reflects the 20^th^ century return interval of spruce budworm outbreaks that occurred every 30–40 years at the landscape scale [31,35,43,54,110]. The similar intervals identified indicate that both proxies detected spruce budworm impacts. A strong lepidopteran scale signal occurred in the latter half of the 20^th^ century but was not observed in the first half. This pattern was evident at most sites, and may be due to little variability in scale accumulations from 1900–1950 which may have been amplified by the interpolation process making it difficult to distinguish peaks from background accumulations, in combination with the shortening of the scale record to match the dendrochronological record (S3 Appendix). Conversely, we observed a strong signal in the dendrochronological data set over the entire study period (1900–2019) for both mean GSI and percent affected. However, the strength, return intervals, and time over which the signal was observed in both the scale and tree-ring records were site dependent. For example, at Site 4 a strong signal is observed in mean GSI and percent affected prior to 1950 but not after 1950, meanwhile we observed a strong signal in the scale record after 1950. Conversely, Site Bois Joli displayed a strong signal in both proxy records throughout the study period.

The cross-wavelet analysis also identified zones of strong significant overlapping signals common to both records at intervals of 16–32 years over the study period (1900–2019), suggesting that both proxy records, relative to one another, detect periods of high spruce budworm impact. However, we observed a difference in the strength of overlapping power when comparing lepidopteran scales to the mean GSI instead of percent affected. Although significant, the weak overlap in power in the mean GSI signal relative to the percent affected signal may be because the accumulation of lepidopteran scales reflects the stand-level phenomenon of insect biomass at a site. Mean GSI on the other hand, using the mean annual ring width of the stand [88], may introduce more tree-level variability, and wavelet analysis may be sensitive to this variability. If this tree-level variability exceeds the background signal generated by the analysis, it will be considered as signal, and although a general pattern may emerge, a fuzzier signal occurs in years when a single tree expresses good growth, and the majority do not. It is also worth acknowledging that lepidopteran scale accumulation interpolation to an annual resolution could have also affected the detected scale-mean GSI signal pair comparison.

Two reasons may explain the stronger overlap in the lepidopteran scales and percent trees affected pair. The first relates to the nature of the paleo proxy. As mentioned above, lepidopteran scales accumulations reflect the insect population in a stand, and not on an individual tree and moreover, scale accumulations reflect moderate and severe defoliation events [65]. The second reason is methodological relating to how the percent affected metric is calculated. As stated earlier, the percent affected metric does not assess the exact magnitude of growth reduction expressed by each tree, it relies on predetermined thresholds to classify individual trees as being defoliated or not and based on this classification determines the proportion of defoliated trees in a stand [50,88]. Additionally, the applied thresholds most likely reflect moderate and severe defoliation. As such, the coarser representation of the spruce budworm’s impact represented by the proportion of affected trees compares similar defoliation impacts and better corresponds to the stand-level phenomenon measured by the scale accumulations resulting in the stronger overlap of high common power. In contrast, the mean GSI measures individual tree-level changes in growth obtaining an average outcome for the stand noting that in this study a non-host correction could not be applied. However, site-level SPEIs from 1901−2011 did not reveal drought conditions (values exceeding −2) during the known outbreak periods (S1 Appendix). Further, correlations and cross-correlations between site-level SPEI and tree-ring chronologies were insignificant (weakly correlated at site Bois Joli) suggesting that growth reductions recorded were due to the defoliation by the spruce budworm (S1 Appendix).

Wavelet coherence revealed a significant correlation over a short time period between the scale and tree-ring records, suggesting that the signals were synchronous. This correlation was typically observed at a periodicity of four years or less, although the periodicity ranged from 0 to 16 years. Here, the return intervals (periodicities), can be interpreted as the time required for the proxies to record the spruce budworm signal. The zero-to-four-year periodicity between the signals likely results from a combination of the time required for scale incorporation into the sediment, potential errors associated with the dating of the sediment core that carry over into the age–depth models for each core [112,123], and the lag of two to four years after the onset of an outbreak for defoliation to become apparent in individual trees [47,48]. However, we also recorded a significant correlation at longer intervals, and the implications of this remain unclear. A significant correlation at an interval ≥16 years was present in the latter half of the 20^th^ century for sites 2, 3, 4, and 8. These zones of high correlation may result from chance, producing a spurious correlation between the proxy records at longer intervals.

Site history, particularly changes to the proportion of host-trees, can influence the spruce budworm signal recorded by the lepidopteran scales and tree-rings producing variable areas of high power and dampen one or both proxy record signals affecting detected correlations. For example, timber harvesting, as observed around lakes 2, 3, and 8 in the latter half of the 20^th^ century (Table 1 and S3 Appendix), has the potential of altering host-tree proportions and influence which individual trees remain on site. A reduction in host-tree proportion limits spruce budworm abundance likely resulting in small populations causing the paleo-proxy to record accumulations that are indistinguishable from background accumulations. Interestingly, even given a low abundance of spruce budworm, the few surviving host-trees left behind from harvesting may record low defoliation or possibly more severe defoliation that does not reflect the budworm population due to a greater density of larvae on a smaller food source [124–126]. In this situation, the proxy records could produce more monotonous or even opposing signals potentially resulting in spurious correlations. For example, the effect of past and current harvesting is well demonstrated within Site 2, as neither individual proxy exhibits a strong signal (zones of significant power). Curiously, the proxies at Site 2 still exhibit a significantly strong correlation, most likely spurious. Analogously, previous fires may also produce spurious correlations at longer return intervals as observed at Site 4 modifying the proportion host-trees in a stand via the stochastic nature of ignitions, burn area, and tree mortality. Finally noting that strong signals at other return intervals may also be a result of mathematical artifacts such that multiples of a particular return interval may be highlighted. In this case, the biologically relevant 32-year interval would be identified along with the mathematically, but not biologically relevant intervals of 8 or 16 years.

Wavelet analysis revealed that sedimentary lepidopteran scale and tree-ring records identify spruce budworm outbreaks (both individually and when compared with each other), and their signals are synchronous. The early part of the statement is supported by the presence of areas of high power (red zones; Fig 1) that may be significant (thick black line; Fig 1) in the individual and cross-wavelet spectra coinciding with the approximately 30-year spruce budworm outbreak cycle (30-year period along the y-axis; Figs 1 and 2). The latter part of the statement is supported by the presence of areas of high significant power within one to four years in the wavelet coherence spectra (Fig 3). The final piece of interpretation involves assessing the phases (arrows in the cross-wavelet and coherence figures; Figs 2 and 3) to characterize the relationship between the two proxies. The average directionality of the arrows indicates that both proxy records agree when large lepidopteran scale accumulations coincide with narrow tree rings or when large lepidopteran scale accumulations coincide with a large proportion of affected trees (Table 2). However, this agreement between proxies at the outbreak level was highly variable suggesting a complex relationship (Table 2).

### Composite chronology comparison

The signal obtained from the composite chronologies was species-dependent. We observed a strong, clear signal in the white spruce composite with distinct periods of high lepidopteran scale accumulations, growth suppression, and large proportions of defoliated trees. This finding agrees with previous studies that demonstrated white spruce to be a good recorder of spruce budworm outbreak events [27,29,30,51], and our recorded periods coincide very well with known outbreak periods of 1912–1929, 1949–1959, 1975–1992 [35,36,44,45]. However, there was a consistent 10-year lag between the lepidopteran scale accumulations and the dendrochronological variables, which could result from the paleo-proxy’s inability to differentiate between periods of no or light defoliation [65] corresponding to low population levels. Scales may not record the entire progression of each outbreak, but record the point at which the insect populations surpass a certain threshold corresponding to periods of moderate or severe defoliation [65] whereas tree-rings may be more sensitive and able to record defoliation at lower insect abundances. Alternatively, the lag may be an artifact of sediment dating errors related to the estimation of background ^210^Pb levels that can result from natural variation in precipitation [127,128], sediment compaction and/or remobilization, and floods events for example [70,129–132]. In this case, the errors would be associated with sediment layers closest to the water-sediment interface that constrain the maximum age (and errors) at the top of the chronology [112]. Additionally, the taphonomical processes that govern incorporation of sedimentary charcoal into lake sediments including bioturbation, erosion re-deposition, and subaqueous slumping [123] also likely affect the incorporation and distribution of lepidopteran scales into the sediment column.

In contrast, the black spruce composite record was not as clear as the white spruce record. We recorded periods of growth suppression around 1915, 1950, and 1980, although the latter two low-growth periods were not very evident, whereas the current outbreak was clear as evidenced by peak scale accumulations ca. 2000–2019 and low-growth. The noisier signal in the black spruce composite record may result from the more variable proportion of balsam fir in the surrounding forest of each site affecting defoliation severity, where defoliation severity in black spruce is greater with an increasing proportion of balsam fir [126,133,134]. Additionally, the historical phenological mismatch between larval emergence and budburst along with cooler temperatures may have limited population build-ups and attenuated defoliation on black spruce [5,11–13,34], ultimately weakening past outbreak signals.

### Future research avenues

Finally, a multi-proxy comparison between tree-rings with other paleo-proxies and between different paleo-proxies could also prove fruitful. For example, a comparison using known, recent outbreaks (approximately last 150 years) between tree-rings and spruce budworm head capsules and frass would help in ameliorating the interpretation of their respective accumulations in the sediment [58,59]. Such a comparison could even be extended over longer time periods using subfossil trees [57]. Similarly, a comparison between tree-rings and other paleo-proxies such as isotopes, and pollen ratios [135] could be investigated. Additionally, utilising a combination of lepidopteran scales, pollen, and macrofossils (both plant and insect) could provide more complete and robust long-term reconstructions. The use of ancient DNA may be a promising avenue of research [136,137] as it has been used to reconstruct faunal [138] and plant diversity [139,140] during the Holocene, but would again need to be compared with other spruce budworm proxies.

## Conclusion

Our study demonstrated that lepidopteran scales and tree ring-derived variables recorded the impact of 20^th^-century spruce budworm outbreaks and that the signals obtained from these proxies were (relatively) synchronous. A key takeaway from this study is that using a multi-proxy approach is essential for reconstructing past disturbances. Comparisons of proxy records can determine whether events are recorded in time and help better interpret the magnitude of a given event. More importantly, multiple lines of evidence provide a more robust interpretation of past disturbance events because of differential recording abilities of the archives and the varying degree of influence by site history. Both lepidopteran scales and tree-ring widths performed well in recording the impacts of the spruce budworm, indicating that both proxies are reliable for longer-term reconstructions, and although scales are a valid long-term proxy, care must be taken in site selection, and sediment sampling to ensure accurate reconstructions. As more lakes are sampled and long-term spruce budworm chronologies are obtained from lepidopteran scales, regional composite scale-tree-ring chronologies will offer the best means of assessing how spruce budworm impacts varied at centennial and millennial scales in the context of past climate change because they reduce variability associated with site history, lake-level taphonomical processes, and help circumvent the inability of every lake to record every outbreak [54,83]. This is especially important as there does not appear to be a particular suite of site properties, based on the current data used in this study, that govern precise and accurate spruce budworm event reconstructions requiring further investigation.

## Supporting information

S1 Appendix S1_Appendix_SPEI_v2.pdf. (PDF)

S2 Appendix S2_Appendix_Lake8_v2.pdf. (PDF)

S3 Appendix S3_Appendix_chronologies_v2.pdf. (PDF)

S4 Dataset Dendrochonology_data. (ZIP)

S5 Dataset GAM_output_used_for_wavelet_analysis. (ZIP)

S6 Dataset Lepidopteran_scale_data. (ZIP)

S7 Dataset Belanger_wavelet_data. (ZIP)

S8 Dataset BoisJoli_wavelet_data. (ZIP)

S9 Dataset Buire_wavelet_data. (ZIP)

S10 Dataset Lake_1_wavelet_data. (ZIP)

S11 Dataset Lake_2_wavelet_data. (ZIP)

S12 Dataset Lake_3_wavelet_data. (ZIP)

S13 Dataset Lake_4_wavelet_data. (ZIP)

S14 Dataset Lake_8_wavelet_data_no_outlier. (ZIP)

S15 Dataset Lake_8_wavelet_data_outlier_data. (ZIP)

S16 Dataset Lake_5_wavelet_data. (ZIP)
