# Supplementary material for: Reconstructing spruce budworm outbreak severity: a comparison of paleoecological and tree-ring signals
Source: PLoS One. 2025 Aug 12;20(8):e0329406. doi: 10.1371/journal.pone.0329406 (PMC12342301; doi:10.1371/journal.pone.0329406)
Supplement: S1 Appendix — (PDF) [file pone.0329406.s001.pdf]

Supplemental material

S1 Appendix. Standardized Precipitation Evapotranspiration Index for the study area

A drought index was used to confirm whether or not growth reductions recorded by tree-rings were due to defoliation by the spruce budworm in the absence of applying a non-host correction. The Standardized Precipitation Evapotranspiration Index (SPEI) is a robust drought index [1] that obtains a monthly water balance by subtracting potential evapotranspiration (determined by temperature) from precipitation [2, 3, 4]. The SPEI is a standardized index such that it has a standard deviation of 1 and an average of 0, and therefore is comparable to other SPEI values at different spatiotemporal scales [2, 3]. This metric accurately reflects streamflow, soil moisture, and the high correlation between tree-ring widths and drought [4]. Values greater than 2 or less than -2 suggest extremely wet or extremely dry conditions respectively, meanwhile values oscillating around 0 are considered normal [2].

We used the Canadian historical 12-month SPEI dataset from 1900-2011 consisting of a 50-km grid covering the entire country made available by Environment and Climate Change Canada [5]. We obtained the monthly SPEI values from the grid cells closest to each respective site. The mean summer (June, July, and August) site-level SPEI values for each year were used because this period coincides with the feeding and dispersal period of the spruce budworm [6-10]. Further, the summer period tends to exhibit the warmest temperatures during which trees are most likely to suffer from reduced growth due to water stress [11-13]. Outbreak periods were defined as occurring from 1912-1929 (O3), 1946-1959 (O2), and 1975-1992 (O1) based on previously published studies [14-17].

The SPEI values do not appear to identify any drought periods during the defined outbreaks (S1 Fig 1). The SPEI during the outbreak periods generally oscillated within a range of values that would be considered normal, although O3 occurred during a period that was slightly drier than normal. The lack of obvious low SPEI values during the outbreak periods suggests that the growth reductions recorded by the cored trees are not due to climate but most likely result from the defoliation by the spruce budworm. This is further supported by the lack of significant correlation between site-level SPEI and ring-width chronologies (S1 Table 1; S1 Fig 2). The presence of a significant weak correlation was found at site Bois Joli suggesting a relationship between site-level SPEI and ring-width chronologies. However, correlation between the chronologies does not preclude the possibility that the spruce budworm was present and impacting tree growth during drier periods. Specifically, drier periods may predispose trees to subsequent defoliation by the budworm [18-20].

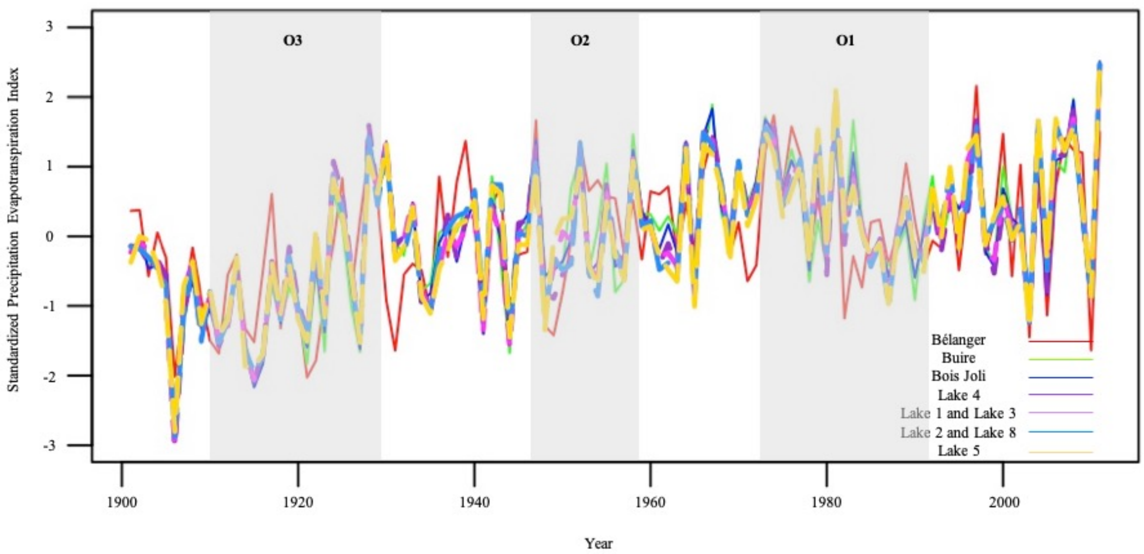

S1 Fig 1. Mean Standardized Precipitation Evapotranspiration Index values for summer months from 1901-2011. The approximate periods of spruce budworm outbreaks are delineated by the grey boxes identified as O3 (1912-1929), O2 (1946-1959), and O1 (1975-1992). SPEI values greater or less than 2 or -2 correspond to extremely wet or extremely periods respectively.

S1 Table 1. Pearson correlations between site-level Standardized Precipitation Evapotranspiration Index and ring-width index chronologies.

Lake	Time span	Degrees of freedom	Pearson's correlation (rho)	p-value
5	1901-2011	109	0.07	0.48
8	1901-2011	109	-0.05	0.63
2	1901-2011	109	-0.09	0.34
1	1901-2011	109	0.06	0.53
3	1901-2011	109	0.08	0.39
4	1922-2011	88	0.13	0.21
Bois Joli	1901-2011	109	*0.20*	*0.04*
Buire	1901-2011	109	0.14	0.14
Bélanger	1911-2011	99	-0.04	0.72

Significant correlations are in *italics* using a significance threshold value of 0.05.

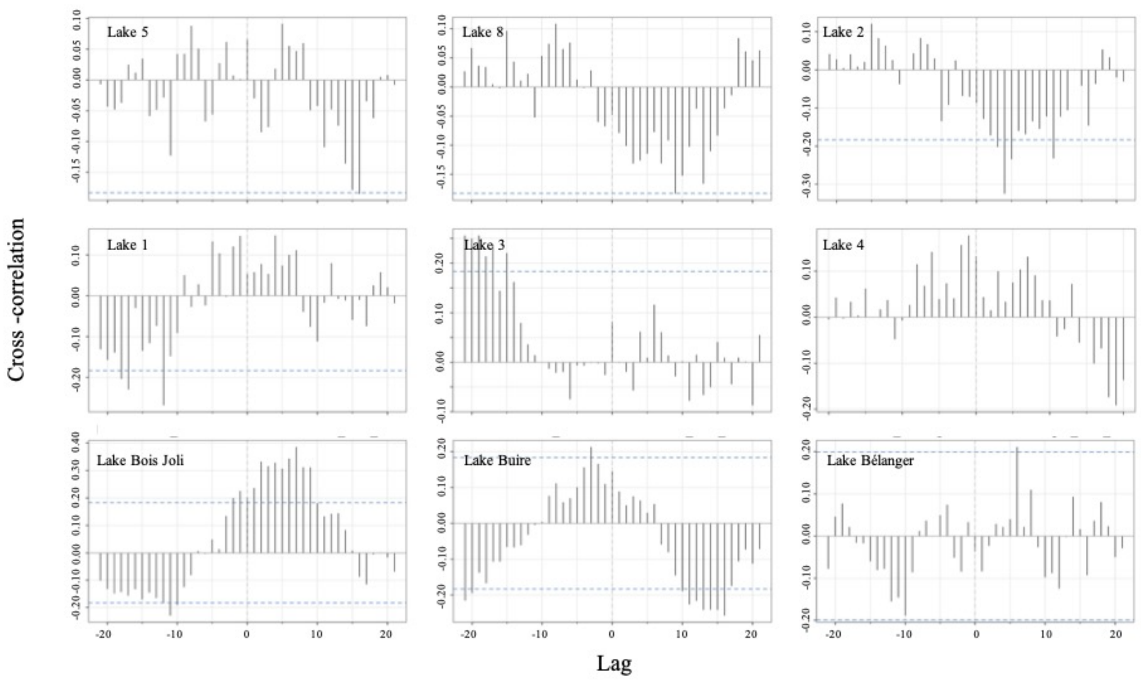

S1 Fig 2. Site-level cross-correlations between Standardize Precipitation Evapotranspiration Index and ring-width index chronologies. Negative lag years suggest that the SPEI chronology *leads* the RWI chronology, meanwhile positive lag years suggests the SPEI chronology *lags* the RWI chronology. Dotted blue lines identify the 95% confidence interval.

References

1. Beguería S, Vicente-Serrano SM, Reig F, and Latorre B. Standardized precipitation evapotranspiration index (SPEI) revisited: parameter fitting, evapotranspiration models, tools, datasets and drought monitoring. Int J Climatol. 2014; 34(10): 3001-3023
2. Vicente-Serrano SM, Beguería S, and López-Moreno JI. A multiscalar drought index sensitive to global warming: the standardized precipitation evapotranspiration index. J Clim. 2010a;23(7):1696-1718
3. Vicente-Serrano SM, Beguería S, López-Moreno JI, Angulo M, and El Kenawy A. A new global 0.5° gridded dataset (1901-2006) of a multiscalar drought index: comparison with current drought index datasets based on the Palmer Drought Severity Index. J. Clim. 2010b; 11(4): 1033-1043
4. Vicente-Serrano SM, Beguería S, Lorenzo-Lacruz J, Camarero JJ, López-Moreno JI, Azorin-Molina C, et al. Performance of drought indices for ecological, agricultural, and hydrological applications. Earth Interact. 2012;16(10): 1-27
5. Environment and Climate Change Canada. Twelve-month historical Standardized Precipitation Evapotranspiration Index values for Canada from 1900-2011; 2015 [accessed June 17 2024]. Database: CSV file [Internet]. Available from: https://open.canada.ca/data/dataset/55fa8f11-aa8c-4232-86da-2e370239e096
6. MacLean DA. Impacts of insect outbreaks on tree mortality, productivity, and stand development. Can Entomol. 2016;148: S138-S159
7. Nealis VG. Comparative ecology of conifer-feeding spruce budworms (Lepidoptera: Tortricidae). Can Entomol. 2016;148: S33-S57
8. Royama T. 1984. Population dynamics of the spruce budworm *Choristoneura fumiferana*. Ecol Monogr.1984;54(4): 429-462
9. MacLean DA. Vulnerability of fir-spruce stands during uncontrolled spruce budworm outbreaks-A review and discussion. For Chron. 1980;56(5): 213-221
10. MacLean DA. 1984. Effects of spruce budworm outbreaks on the productivity and stability of balsam fir forests. For Chron. 1984;60(5): 273-279

11. Cooke ER, Seager R, Cane MA, and Stahle DW. North American drought: reconstructions, causes, and consequences. Earth Sci Rev. 2007;81(1-2): 93-134
12. D'Orangeville L, Maxwell J, Kneeshaw D, Pederson N, Duchesne L, Logan T et al. Drought timing and local climate determine the sensitivity of eastern temperate forests to drought. Glob Chang Biol. 2018;24(6): 2339-2351
13. Au TF, Maxwell JT, Novick KA, Robeson SM, Warner SM, Lockwood BR, et al. Demographic shifts in eastern US forests increase the impact of late-season drought in forest growth. Ecography. 2020;43(10): 1475-1486
14. Boulanger Y, and Arseneault D. Spruce budworm outbreaks in eastern Québec over the last 450 years. Can J For Res. 2004;34(5): 1035-1043
15. Gray DR, Régnière J, Boulet B. Analysis and use of historical patterns of spruce budworm defoliation to forecast outbreak patterns in Québec. For Ecol Manage. 2000;127(1-3): 217-231
16. Morin H, and Laprise D. 1990. Histoire récente des épidémies de la Tordeuse des bourgeons de l'épinette au nord du lac-Saint-Jean (Québec): une analyse dendrochronologique. Can J For Res. 1990;20(1): 1-8
17. Morin H. Dynamics of balsam fir forests in relation to spruce budworm outbreaks in the boreal zone of Québec. Can J For Res. 1994;24(4): 730-741
18. Candau, J-N, and Fleming RA. Landscape-scale spatial distribution of spruce budworm defoliation in relation to bioclimatic conditions. Can. J. For. Res. 2005;35: 2218-2235
19. Greenbank DO. The role of climate and dispersal in the initiation of outbreaks of the spruce budworm in New Brunswick. Can J Zool. 1956;34(5): 453-476
20. Pilon JG, and Blais JR. Weather and outbreaks of the spruce budworm in the province of Quebec from 1939 to 1956. Can Entomol. 1961; 93(2): 118-123
