# Supplementary material for: Reconstructing spruce budworm outbreak severity: a comparison of paleoecological and tree-ring signals
Source: PLoS One. 2025 Aug 12;20(8):e0329406. doi: 10.1371/journal.pone.0329406 (PMC12342301; doi:10.1371/journal.pone.0329406)
Supplement: S2 Appendix — (PDF) [file pone.0329406.s002.pdf]

Supplemental material

S2 Appendix. Effects of the removal and replacement of the outlier data point in the Lake 8 mean GSI series

We removed and replaced an outlier from the mean GSI series, as it blurred the observed signal with downstream effects in wavelet analysis. We applied an identical procedure with the outlier and the imputed data point that replaced the outlier as described in the methods section. Retention of the outlying point for the year 1952 resulted in a linear mean GSI signal, when the mean GSI signal was not linear and actually decreased—presumably because of defoliation over the course of the ca. 1950 spruce budworm outbreak —as observed in other dendrochronological studies (S2 Fig1a) [1]. To obtain a more representative signal, we removed this outlier. However, to conduct wavelet analysis, a constant time-step is required; therefore, we interpolated a data point to replace the outlier. We believe that the resulting modeled signal better reflected the trend in mean GSI (S2 Fig 1b).

The retention or replacement of the outlier subsequently affected the wavelet analysis conducted on the scale and tree-ring records. The power spectra of the mean GSI differed markedly (S2 Fig 2). The power spectrum with the retained outlier failed to detect any signal relative to red noise (S2 Fig 2a), whereas the power spectrum of the mean GSI with the imputed data point produced an area of significant power at a periodicity of about 32 years over the course of the middle to latter portion of the 20th century (S2 Fig 2b). Similarly, the effect of the retained outlier cascaded into the cross-wavelet and wavelet coherence analyses. The influence of the retained outlier did not markedly change the outcome of the cross-wavelet analysis in terms of strength of areas of common power. Nonetheless, the zones of statistical significance did in fact vary as did the associated phases (S2 Fig 3). The differences in wavelet coherence analysis were less subtle as different areas of significantly high correlation were identified, depending on whether we retained or replaced the outlier (S2 Fig 4). Furthermore, the associated phases in these areas of significant high correlation also differed (S2 Fig 4). We believe that the model using the replaced data point in the mean GSI is more representative of the data, and thus used this imputed data point for our analyses.

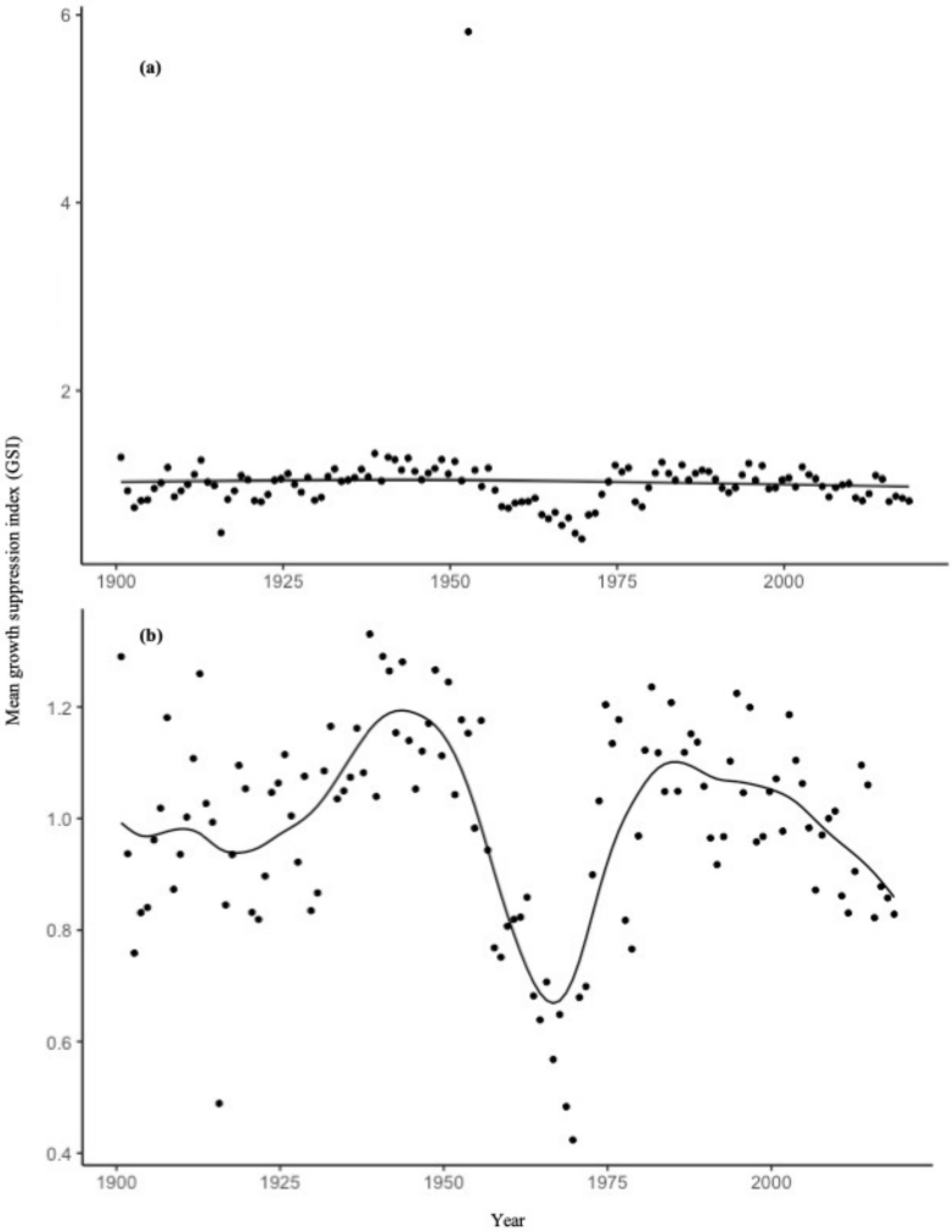

S2 Fig 1. The fitted GAM to the mean GSI of Lake 8. The series is presented (A) with the outlier and (B) with the interpolated data point.

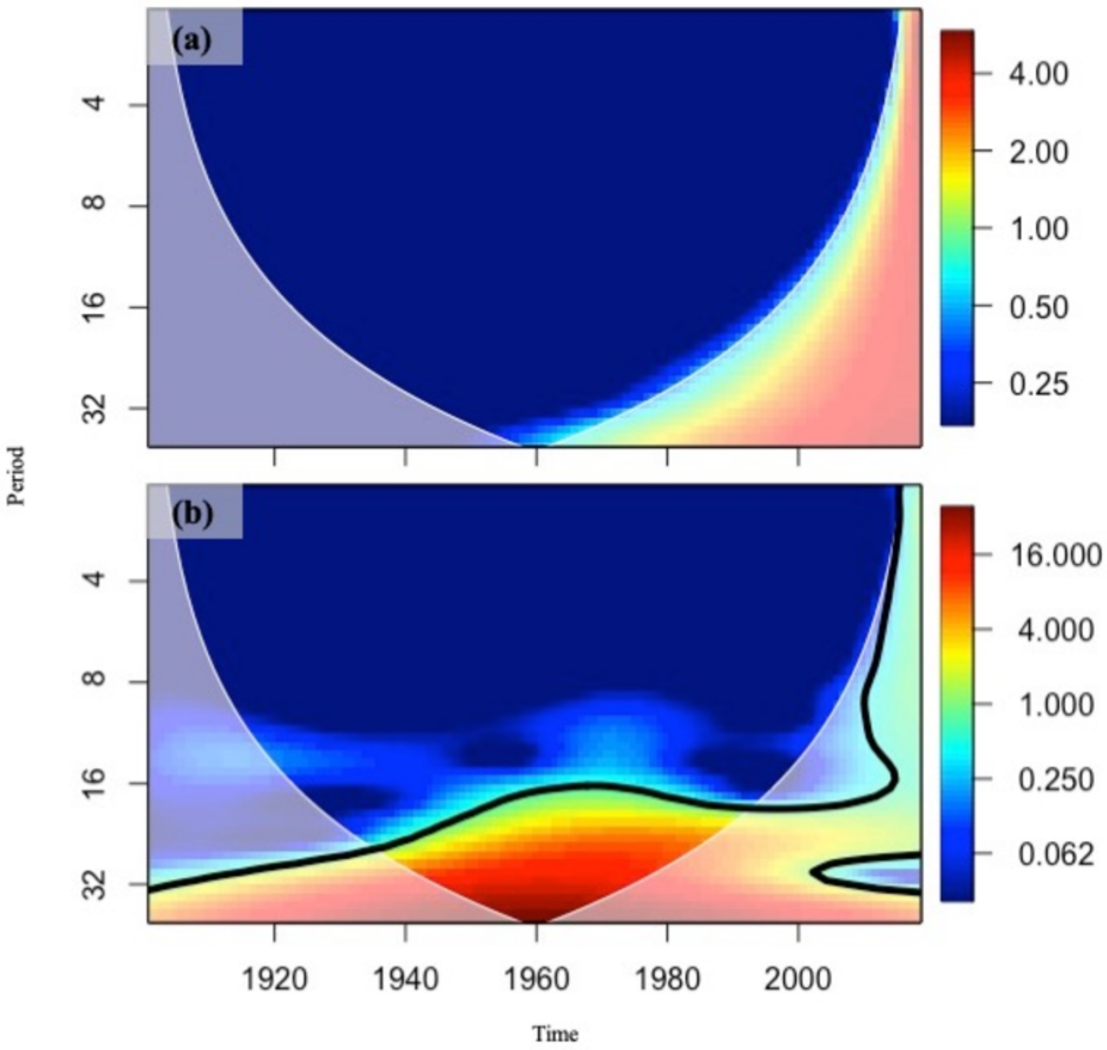

S2 Fig 2. Lake 8's power spectra for the mean GSI series. The series is presented (A) with the outlier and (B) with the interpolated data point**.** Warmer colours (red, orange, yellow) indicate high power relative to red noise, an autoregressive process with lag 1, whereas cooler colours suggest weaker power [2]. Statistically significant zones of power were determined using a χ^2 test ($p<0.05$) and are delineated by a thick black line [2]. The light grey shading delineates the recommended zone of interpretation, i.e., 'cone of influence' [3].

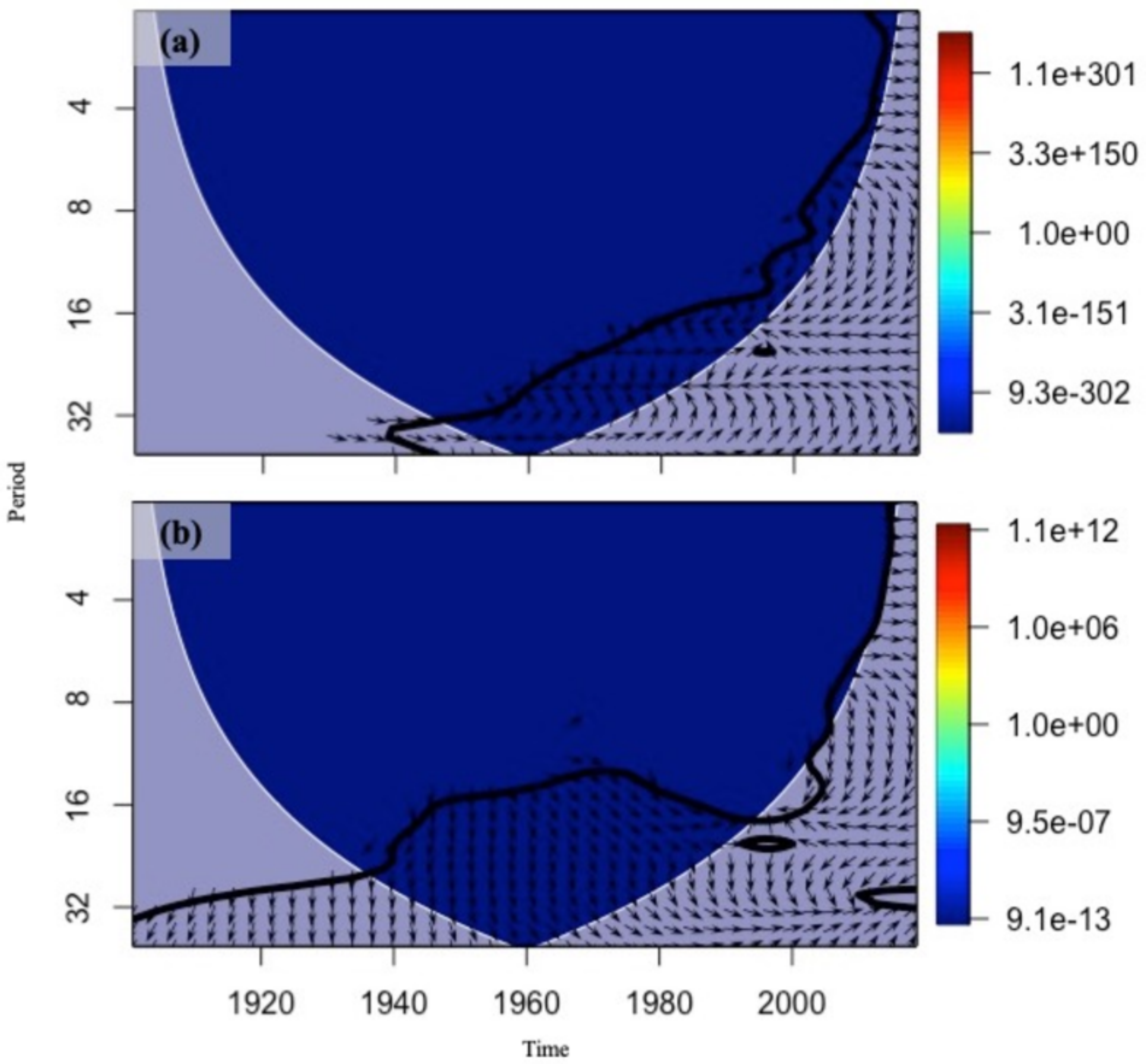

S2 Fig 3. Lake 8's cross-wavelet analysis between the lepidopteran scale and mean GSI series. The result of the analysis **(**A) with the outlier, and (B) with the interpolated data point. Warmer colours (red, orange, yellow) indicate high overlapping power relative to red noise, an autoregressive process with lag 1, whereas cooler colours suggest weaker overlapping power [2]. Statistically significant zones of power were determined using a χ^2 test ($p<0.05$) and are delineated by a thick black line [2]. The arrows in the areas of statistical significance specify the type of association. Arrows pointing left indicate the signals are anti-phase, where a peak of one signal lines up with a trough of the other signal. Arrows pointing right suggest signals are in-phase, where peaks and troughs of both signals line up. Downward arrows indicate that the scale record leads the tree-ring record by $\frac{\pi}{2}$, whereas upward arrows indicate the opposite. The light grey shading delineates the recommended zone of interpretation, i.e., 'cone of influence' [3].

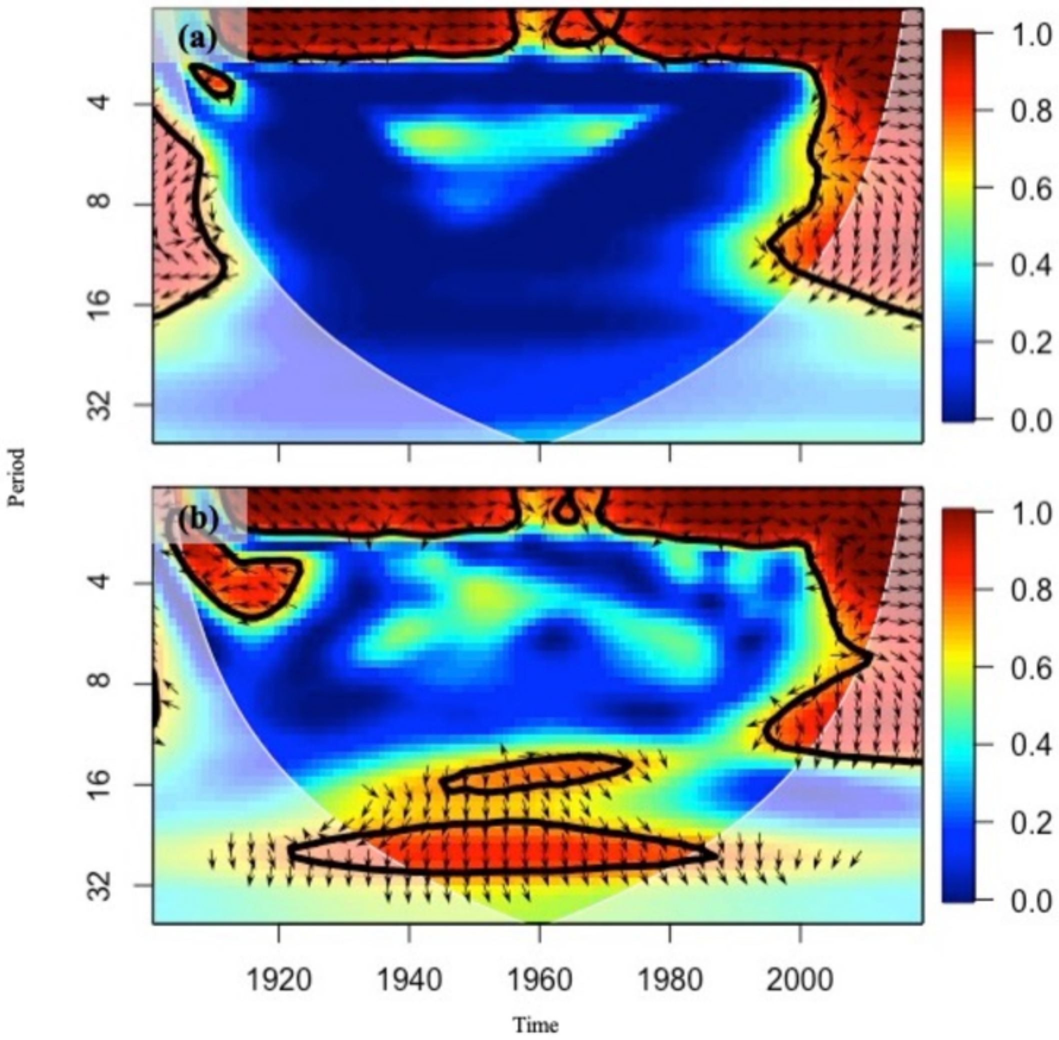

S2 Fig 4. Lake 8's wavelet coherence analysis between the lepidopteran scale and mean GSI series. The result of the analysis (A) with the outlier, and (B) with the interpolated data point. Warmer colours (red, orange, yellow) indicate high correlation, relative to red noise, autoregressive process of lag 1, whereas cooler colours represent weaker correlations [2]. Statistically significant zones were determined using a χ^2 test ($p<0.05$) and are delineated by a thick black line [2]. The arrows in areas of statistical significance specify the type of association. Arrows pointing left indicate the signals are anti-phase, where a peak of one signal lines up with a trough of the other signal. Arrows pointing right suggest signals are in-phase, where peaks and troughs of both signals line up. Downward arrows indicate that the scale record leads the tree-ring record by $\frac{\pi}{2}$, whereas upward arrows indicate the opposite. The light grey shading delineates the recommended zone of interpretation, i.e., 'cone of influence' [3].

References

1. Boulanger Y, Arseneault D, Morin H, Jardon Y, Bertrand P, and Dagneau C. Dendrochronological reconstruction of spruce budworm (*Choristoneura fumiferana*) outbreaks in southern Quebec for the last 400 years. Can J For Res. 2012;42: 1264-1276
2. Torrence C, and Compo GP. 1998. A practical guide to wavelet analysis. Bull Am Meteorol Soc. 1998;79(1): 61-78
3. Cazelles B, Chavez M, Berteaux D, Ménard F, Vik JO, Jenouvrier S, et al. Wavelet analysis of ecological time series. Oecologia. 2008;156(2): 287-304
