# Supplementary material for: Reconstructing spruce budworm outbreak severity: a comparison of paleoecological and tree-ring signals
Source: PLoS One. 2025 Aug 12;20(8):e0329406. doi: 10.1371/journal.pone.0329406 (PMC12342301; doi:10.1371/journal.pone.0329406)
Supplement: S3 Appendix — (PDF) [file pone.0329406.s003.pdf]

Supplemental material

S3 Appendix. Lepidopteran scales and tree-ring chronologies used for wavelet analysis

The full surface sediment chronologies for the lakes of all sites are presented (S3 Fig 1) along with the corresponding sediment and tree-ring chronologies used for the wavelet analysis for each individual site (S3 Fig 2–10). The temporal interval of lepidopteran scale accumulation analysis was from 1900 AD-2019 (S3 Fig 1) to match the time interval covered by the tree-ring chronologies.

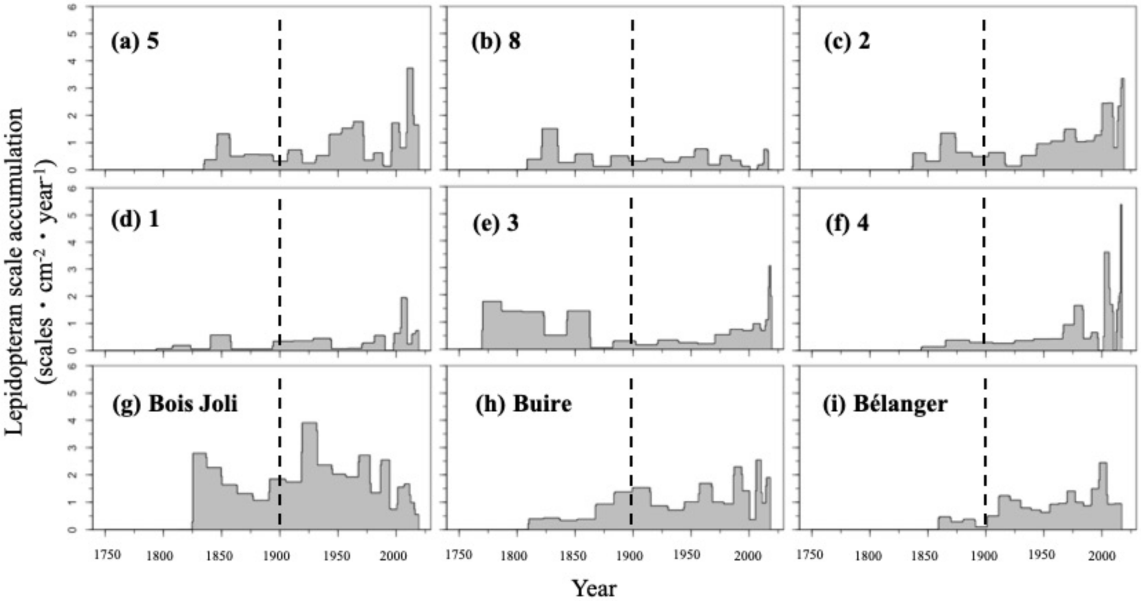

S3 Fig 1. The lepidopteran scale accumulation rate and annually interpolated lepidopteran scale accumulations. The grey bars depict lepidopteran scale accumulations with the continuous black line delineating these bars representing the respective annually interpolated accumulation rates for lakes (A) 5, (B) 8, (C) 2, (D) 1, (E) 3, (F) 4, (G) Bois Joli, (H) Buire, and (I) Bélanger. Accumulation rates to the right of the vertical dotted line (1900 AD) were analyzed in the study, whereas scale accumulations before 1900 AD were not.

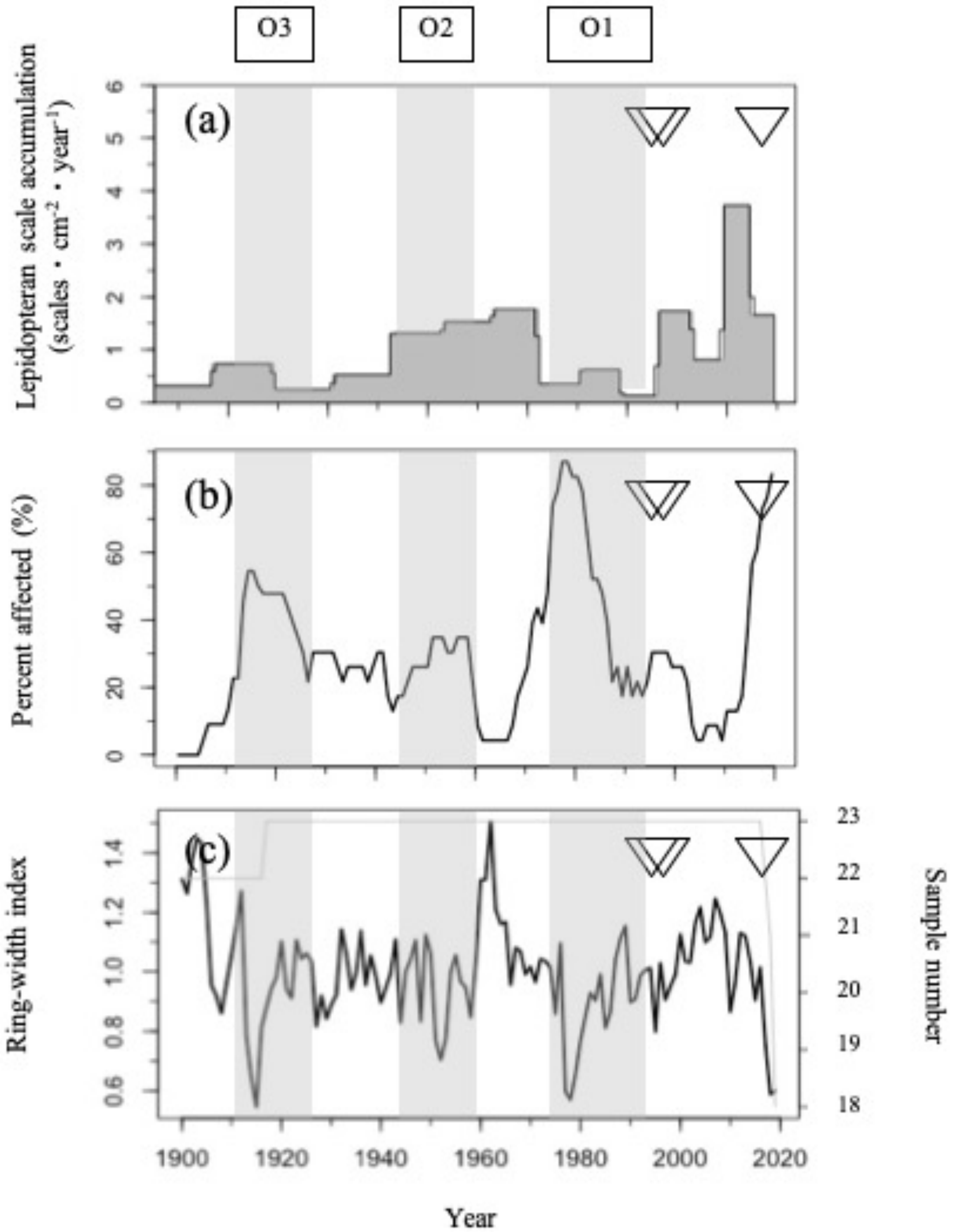

S3 Fig 2. Recorded impacts of the spruce budworm at Site 5. The (A) lepidopteran scale accumulation rate (dark grey bars) and annually interpolated accumulation rate (black line delineating grey bars), (B) the proportion of defoliated trees, and (C) ring-width index (black line), and sample number (grey line) for the expressed population signal (EPS) chronology from 1900-2019. The white triangles represent the approximate dates of known recorded timber harvesting events within a 200 m radius around the lake on the basis of available forest inventory data. O3, O2, and O1 delineate the periods of known spruce budworm outbreaks in the 20th century corresponding to 1912-1929, 1946-1959, and 1975-1992 respectively [1-3].

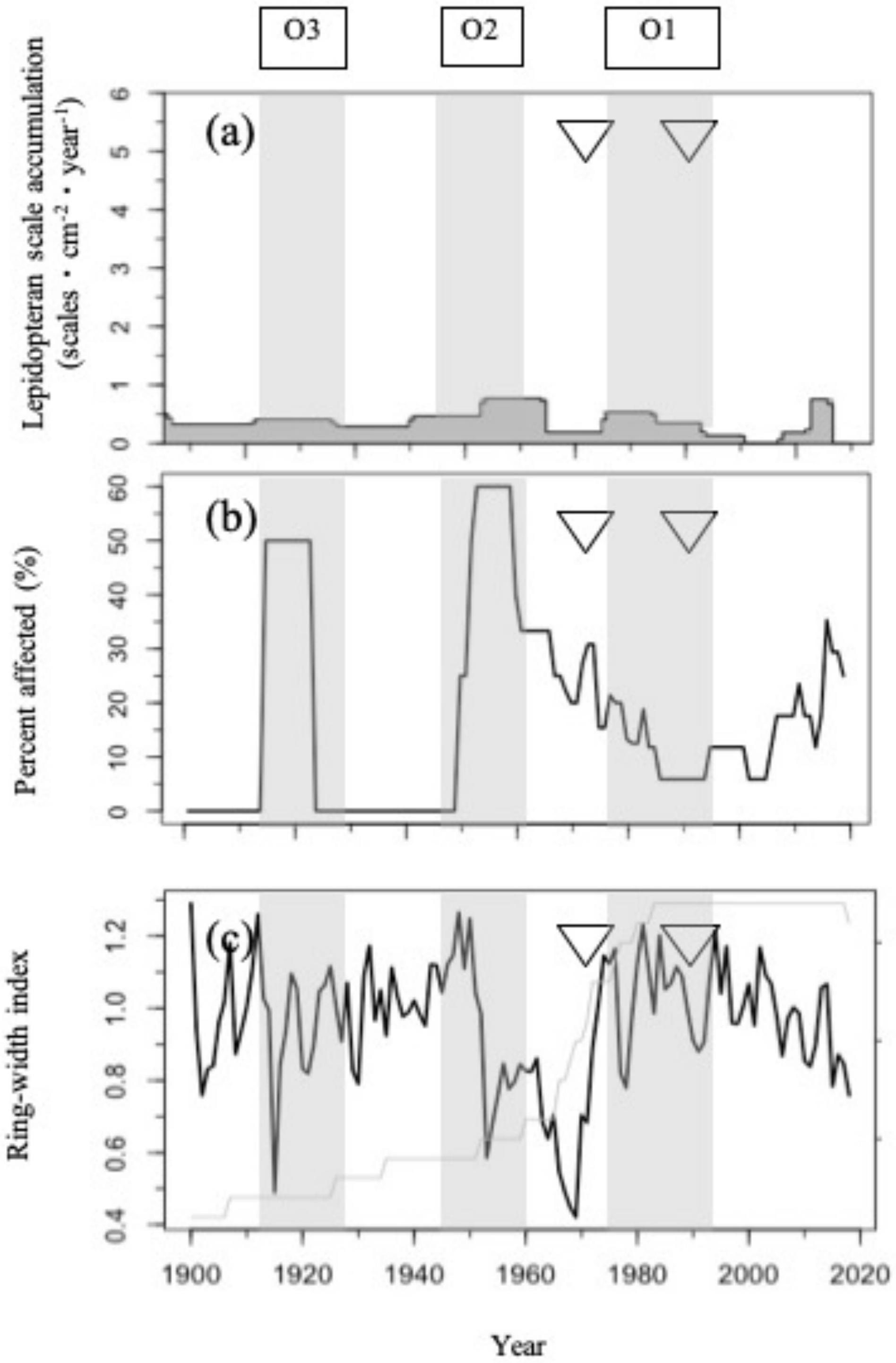

S3 Fig 3. Recorded impacts of the spruce budworm at Site 8. The (A) lepidopteran scale accumulation rate (dark grey bars) and annually interpolated accumulation rate (black line delineating grey bars), (B) the proportion of defoliated trees, and (C) ring-width index (black line), and sample number (grey line) for the expressed population signal (EPS) chronology from 1900-2019. The white triangles represent the approximate dates of known recorded timber harvesting events within a 200 m radius around the lake on the basis of available forest inventory data. O3, O2, and O1 delineate the periods of known spruce budworm outbreaks in the 20th century corresponding to 1912-1929, 1946-1959, and 1975-1992 respectively [1-3].

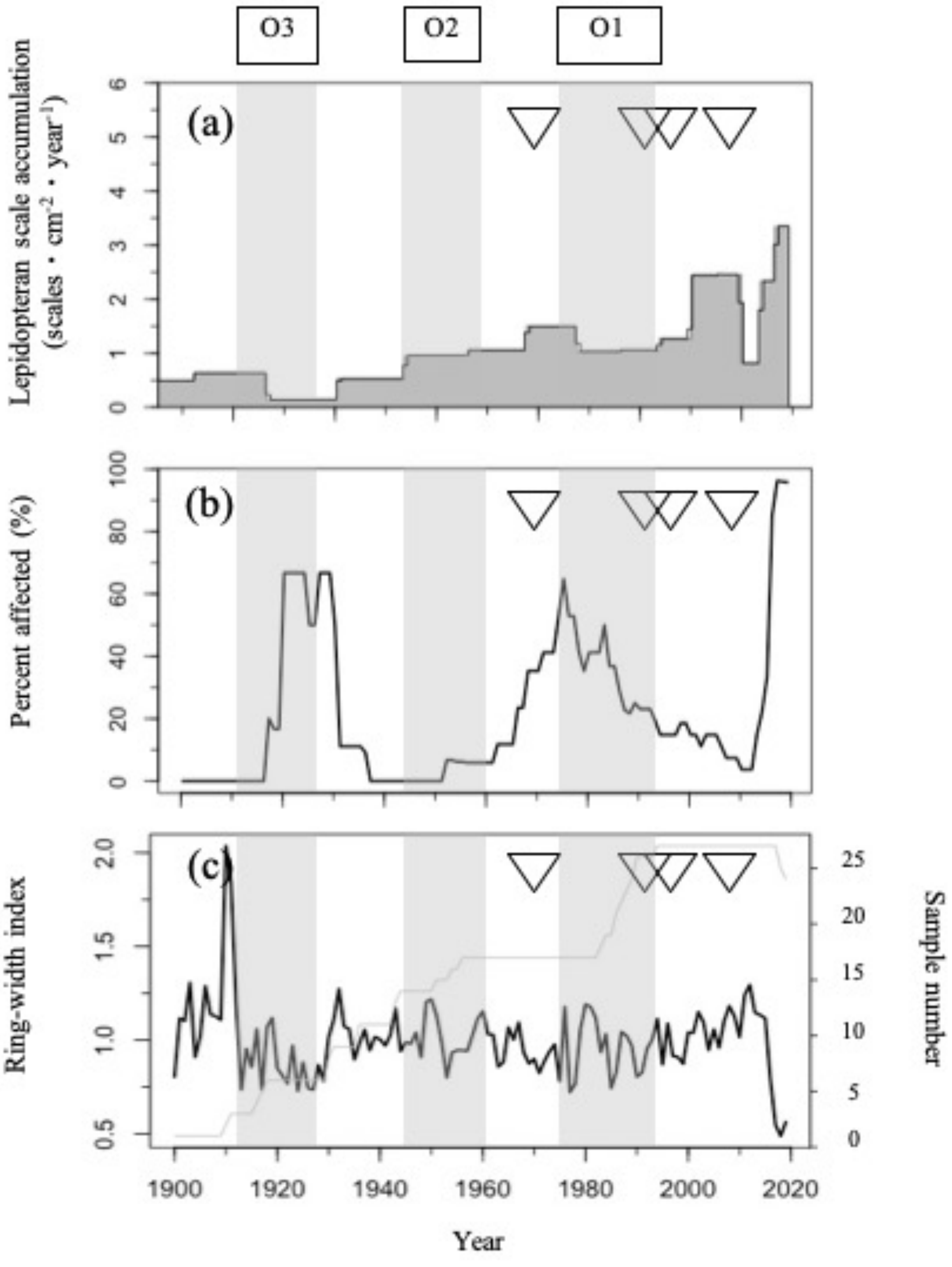

S3 Fig 4. Recorded impacts of the spruce budworm at Site 2. The (A) lepidopteran scale accumulation rate (dark grey bars) and annually interpolated accumulation rate (black line delineating grey bars), (B) the proportion of defoliated trees, and (C) ring-width index (black line), and sample number (grey line) for the expressed population signal (EPS) chronology from 1900-2019. The white triangles represent the approximate dates of known recorded timber harvesting events within a 200 m radius around the lake on the basis of available forest inventory data. O3, O2, and O1 delineate the periods of known spruce budworm outbreaks in the 20th century corresponding to 1912-1929, 1946-1959, and 1975-1992 respectively [1-3].

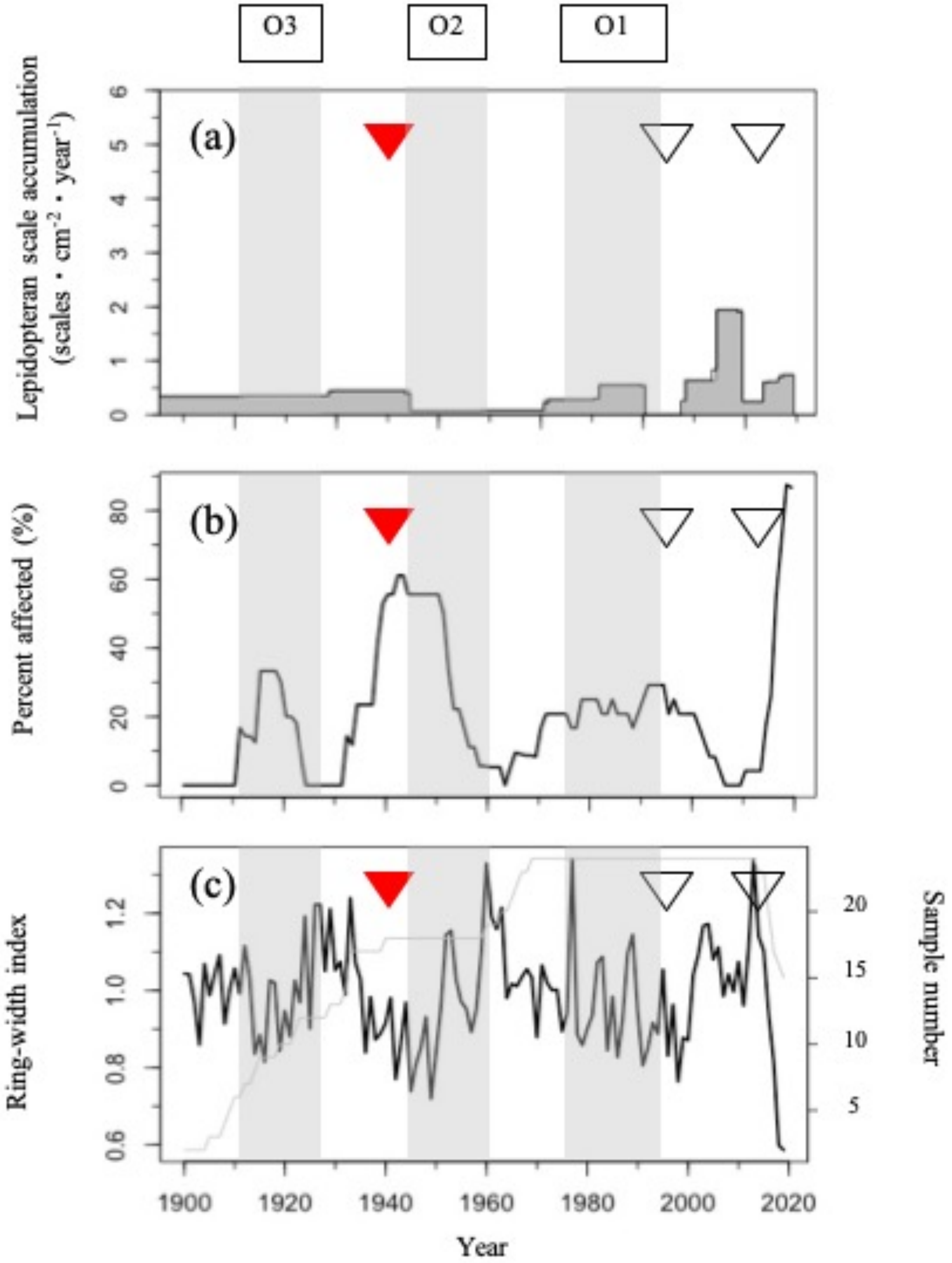

S3 Fig 5. Recorded impacts of the spruce budworm at Site 1. The (A) lepidopteran scale accumulation rate (dark grey bars) and annually interpolated accumulation rate (black line delineating grey bars), (B) the proportion of defoliated trees, and (C) ring-width index (black line), and sample number (grey line) for the expressed population signal (EPS) chronology from 1900-2019. The white and red triangles represent the approximate dates of known recorded timber harvesting, and wildfire events, respectively, within a 200 m radius around the lake on the basis of available forest inventory data. O3, O2, and O1 delineate the periods of known spruce budworm outbreaks in the 20th century corresponding to 1912-1929, 1946-1959, and 1975-1992 respectively [1-3].

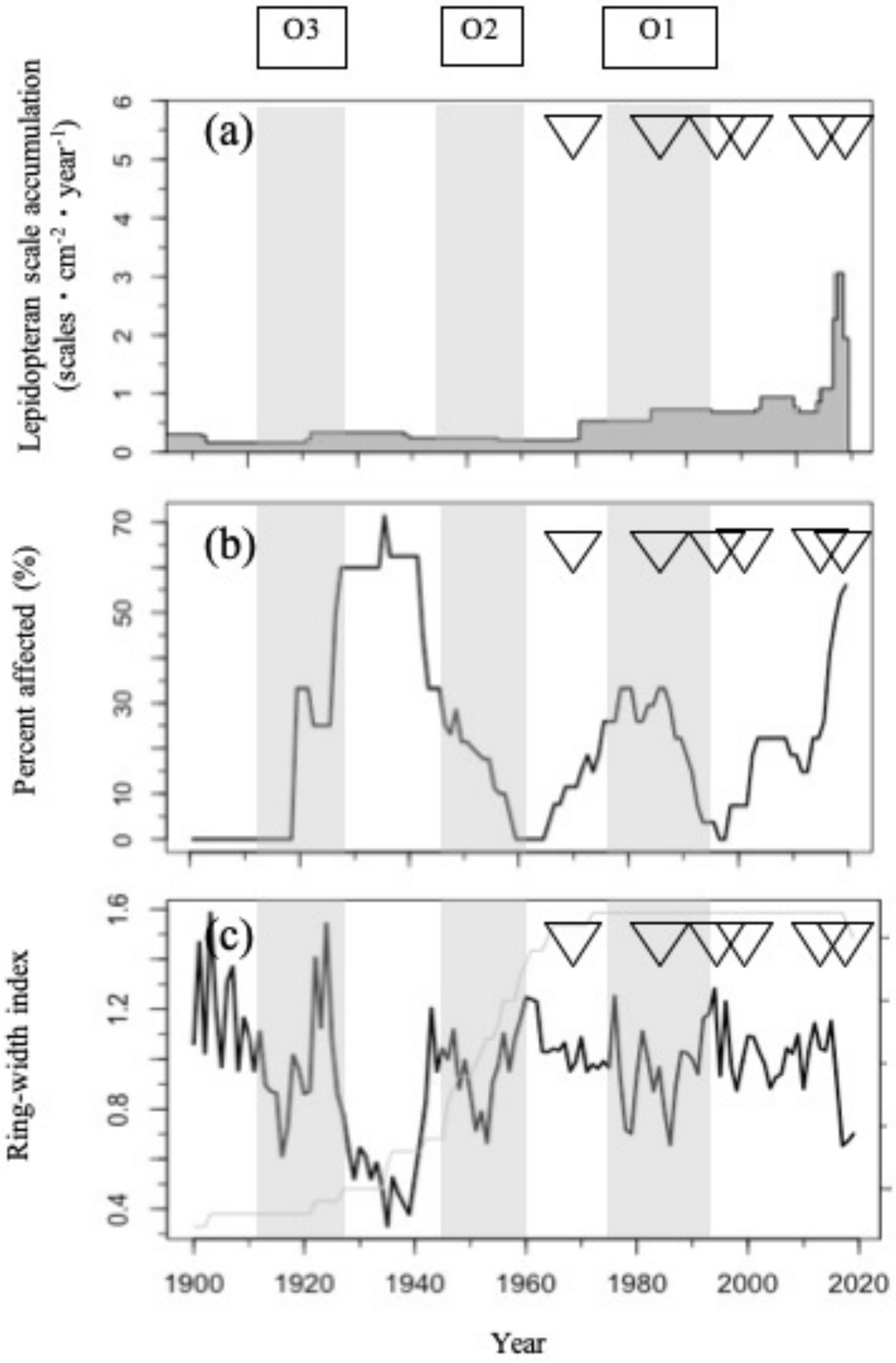

S3 Fig 6. Recorded impacts of the spruce budworm at Site 3. The (A) lepidopteran scale accumulation rate (dark grey bars) and annually interpolated accumulation rate (black line delineating grey bars), (B) the proportion of defoliated trees, and (C) ring-width index (black line), and sample number (grey line) for the expressed population signal (EPS) chronology from 1900-2019. The white triangles represent the approximate dates of known recorded timber harvesting events within a 200 m radius around the lake on the basis of available forest inventory data. O3, O2, and O1 delineate the periods of known spruce budworm outbreaks in the 20th century corresponding to 1912-1929, 1946-1959, and 1975-1992 respectively [1-3].

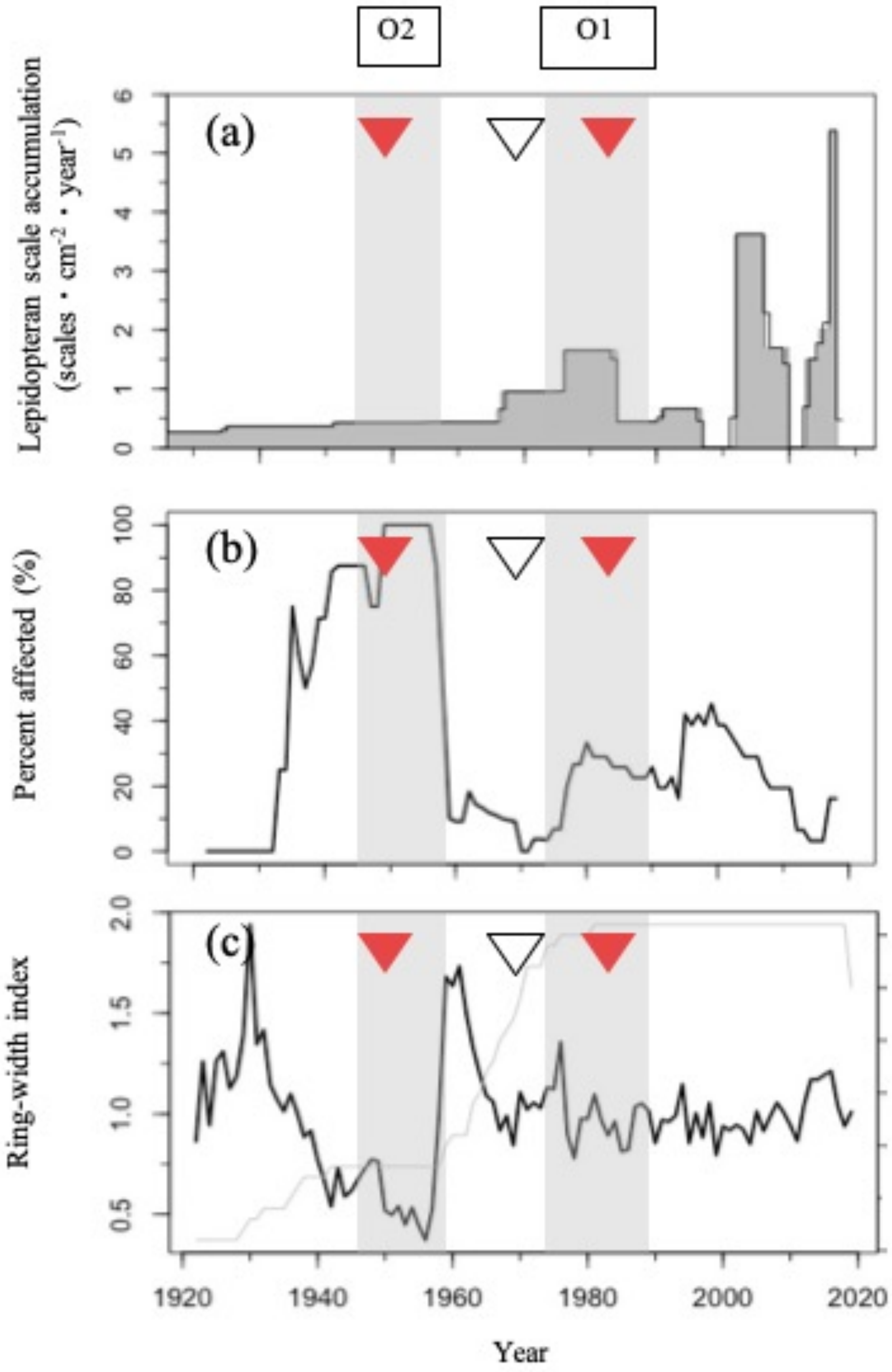

S3 Fig 7. Recorded impacts of the spruce budworm at Site 4. The (A) lepidopteran scale accumulation rate (dark grey bars) and annually interpolated accumulation rate (black line delineating grey bars), (B) the proportion of defoliated trees, and (C) ring-width index (black line), and sample number (grey line) for the expressed population signal (EPS) chronology from 1900-2019. The white and red triangles represent the approximate dates of known recorded timber harvesting, and wildfire events, respectively, within a 200 m radius around the lake on the basis of available forest inventory data. O3, O2, and O1 delineate the periods of known spruce budworm outbreaks in the 20th century corresponding to 1912-1929, 1946-1959, and 1975-1992 respectively [1-3].

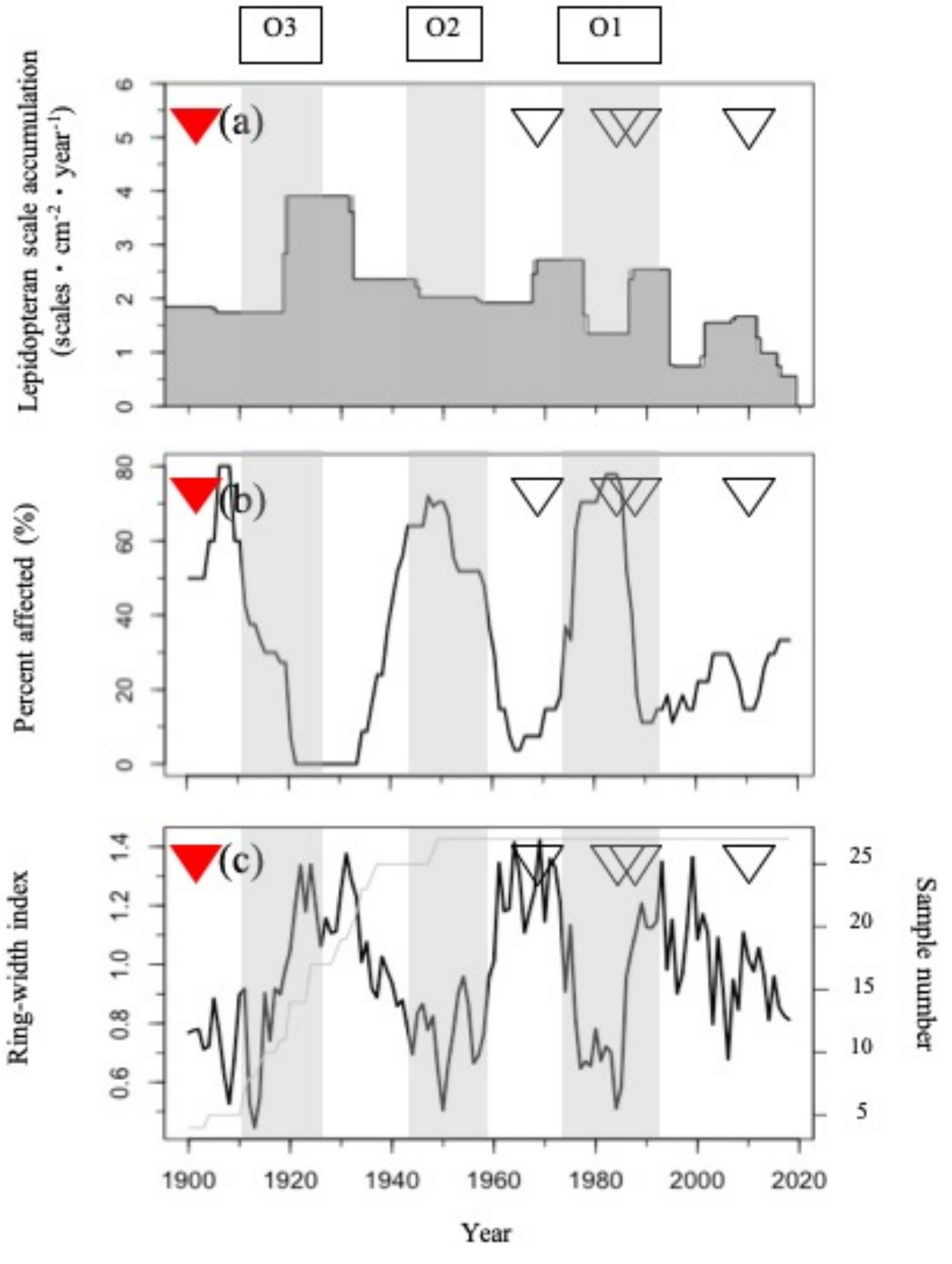

S3 Fig 8. Recorded impacts of the spruce budworm at Site Bois Joli. The (A) lepidopteran scale accumulation rate (dark grey bars) and annually interpolated accumulation rate (black line delineating grey bars), (B) the proportion of defoliated trees, and (C) ring-width index (black line), and sample number (grey line) for the expressed population signal (EPS) chronology from 1900-2019. The white and red triangles represent the approximate dates of known recorded timber harvesting, and wildfire events, respectively, within a 200 m radius around the lake on the basis of available forest inventory data. O3, O2, and O1 delineate the periods of known spruce budworm outbreaks in the 20th century corresponding to 1912-1929, 1946-1959, and 1975-1992 respectively [1-3].

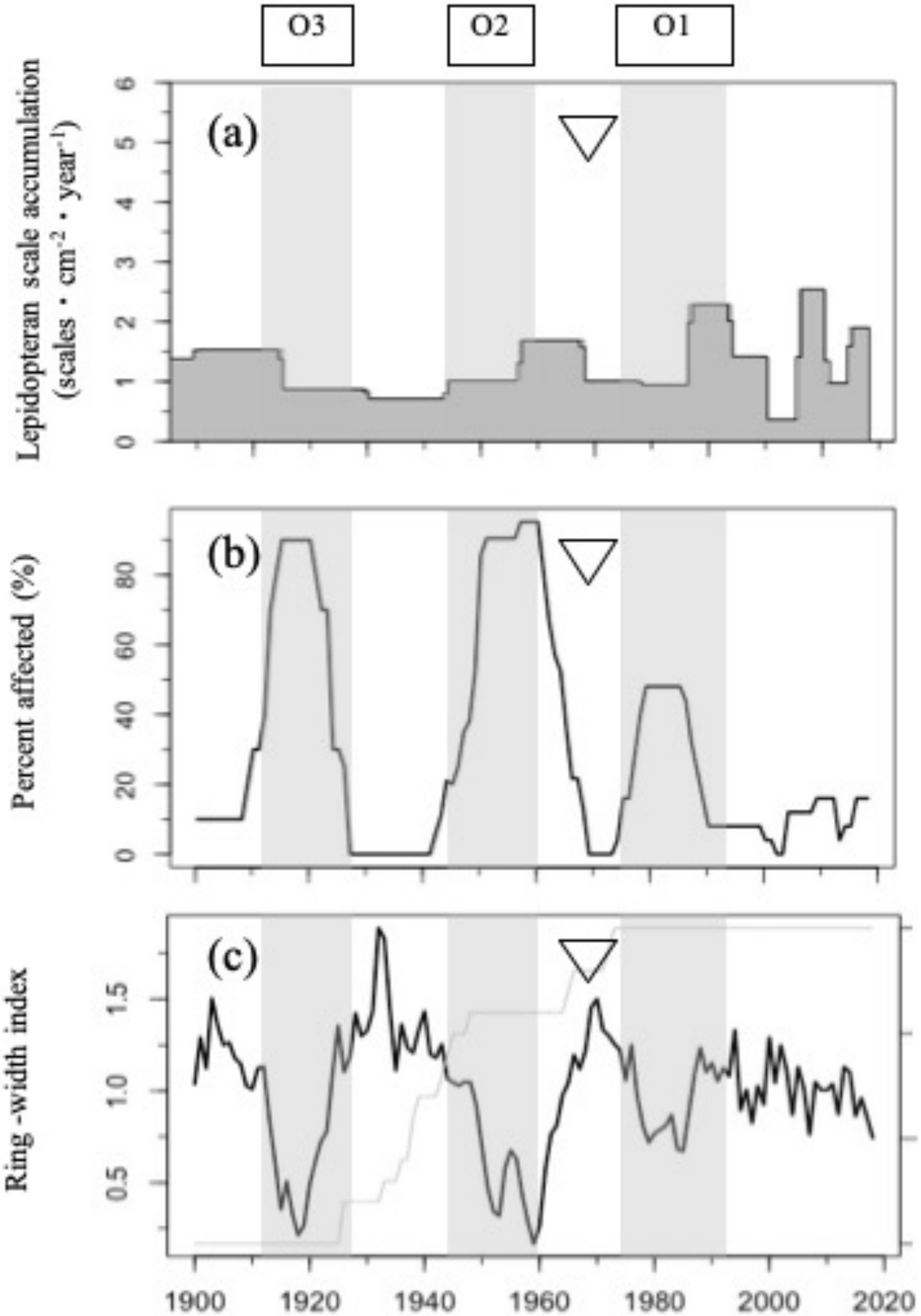

S3 Fig 9. Recorded impacts of the spruce budworm at Site Buire. The (A) lepidopteran scale accumulation rate (dark grey bars) and annually interpolated accumulation rate (black line delineating grey bars), (B) the proportion of defoliated trees, and (C) ring-width index (black line), and sample number (grey line) for the expressed population signal (EPS) chronology from 1900-2019. The white triangles represent the approximate dates of known recorded timber harvesting events within a 200 m radius around the lake on the basis of available forest inventory data. O3, O2, and O1 delineate the periods of known spruce budworm outbreaks in the 20th century corresponding to 1912-1929, 1946-1959, and 1975-1992 respectively [1-3].

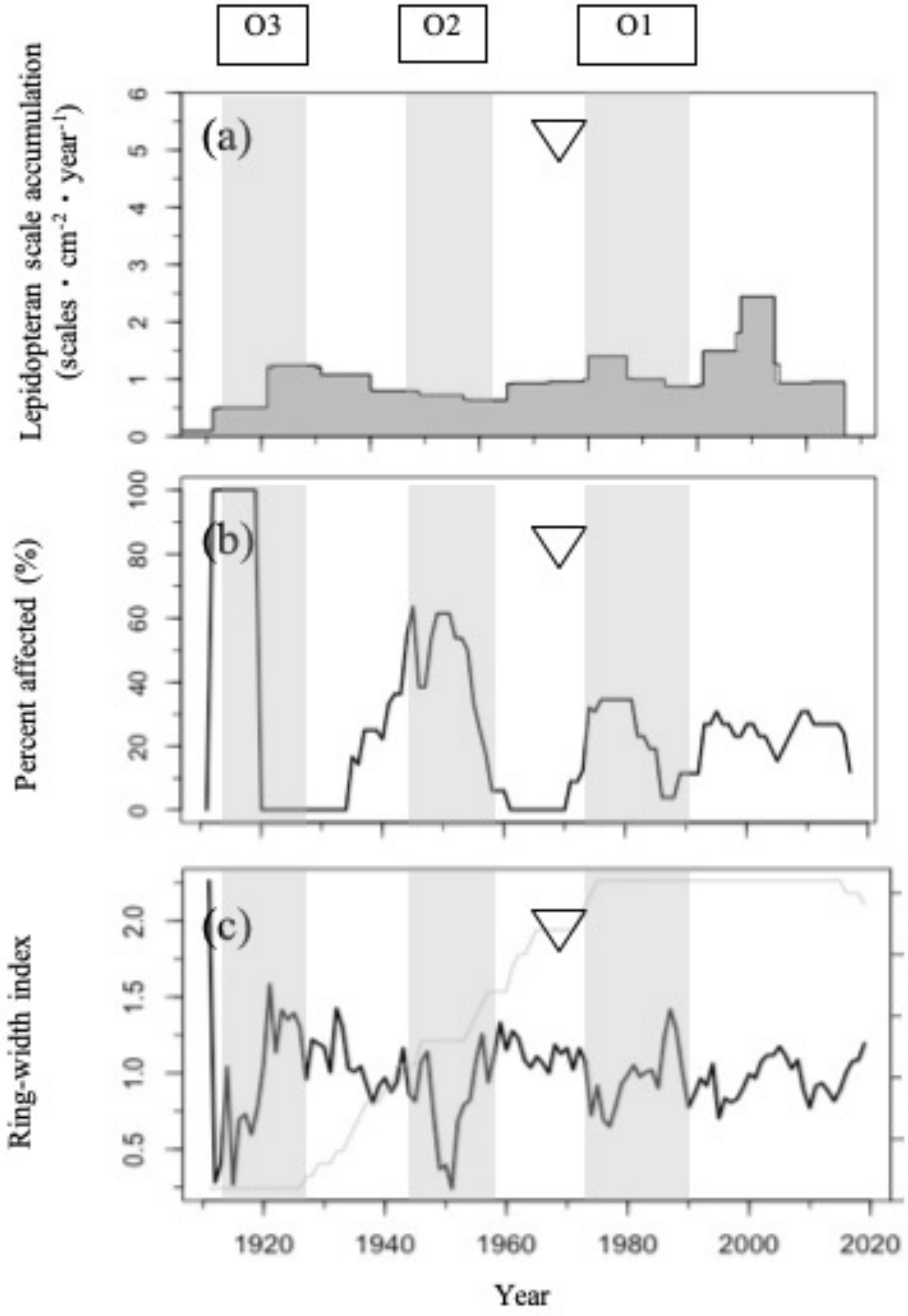

S3 Fig 10. Recorded impacts of the spruce budworm at Site Bélanger. The (A) lepidopteran scale accumulation rate (dark grey bars) and annually interpolated accumulation rate (black line delineating grey bars), (B) the proportion of defoliated trees, and (C) ring-width index (black line), and sample number (grey line) for the expressed population signal (EPS) chronology from 1900-2019. The white triangles represent the approximate dates of known recorded timber harvesting events within a 200 m radius around the lake on the basis of available forest inventory data. O3, O2, and O1 delineate the periods of known spruce budworm outbreaks in the 20th century corresponding to 1912-1929, 1946-1959, and 1975-1992 respectively [1-3].

References

1. Boulanger Y, and Arseneault D. Spruce budworm outbreaks in eastern Québec over the last 450 years. Can J For Res. 2004;34(5): 1035-1043
2. Boulanger Y, Arseneault D, Morin H, Jardon Y, Bertrand P, and Dagneau C. Dendrochronological reconstruction of spruce budworm (*Choristoneura fumiferana*) outbreaks in southern Quebec for the last 400 years. Can J For Res. 2012;42: 1264-1276
3. Morin H, and Laprise D. 1990. Histoire récente des épidémies de la Tordeuse des bourgeons de l'épinette au nord du lac-Saint-Jean (Québec): une analyse dendrochronologique. Can J For Res. 1990;20(1): 1-8
